# Biological therapy in systemic lupus erythematosus, antiphospholipid syndrome, and Sjögren’s syndrome: evidence- and practice-based guidance

**DOI:** 10.3389/fimmu.2023.1117699

**Published:** 2023-04-17

**Authors:** António Marinho, José Delgado Alves, Jorge Fortuna, Raquel Faria, Isabel Almeida, Glória Alves, João Araújo Correia, Ana Campar, Mariana Brandão, Jorge Crespo, Daniela Marado, João Matos-Costa, Susana Oliveira, Fernando Salvador, Lelita Santos, Fátima Silva, Milene Fernandes, Carlos Vasconcelos

**Affiliations:** ^1^ Unidade de Imunologia Clínica, Centro Hospitalar Universitário do Porto, Porto, Portugal; ^2^ UMIB - Unidade Multidisciplinar de Investigação Biomédica, ICBAS - Instituto de Ciências Biomédicas Abel Salazar, Universidade do Porto, Porto, Portugal; ^3^ Systemic Autoimmune Diseases Unit, Hospital Prof. Doutor Fernando Fonseca, Amadora, Portugal; ^4^ Immune Response and Vascular Disease Unit - iNOVA4Health, NOVA Medical School, Lisboa, Portugal; ^5^ Serviço de Medicina Interna, Departamento de Medicina, Centro Hospitalar Universitário de Coimbra, Coimbra, Portugal; ^6^ Serviço de Medicina Interna, Hospital da Senhora da Oliveira - Centro Hospitalar Alto Ave, Guimarães, Portugal; ^7^ Serviço de Medicina Interna, Centro Hospitalar Universitário do Porto, Porto, Portugal; ^8^ Serviço de Medicina Interna, Hospital Distrital de Santarém, Santarém, Portugal; ^9^ Unidade de Doenças Autoimunes, Serviço de Medicina Interna, Centro Hospitalar de Trás-os-Montes e Alto Douro, Vila Real, Portugal; ^10^ Faculdade de Medicina, Universidade de Coimbra, Coimbra, Portugal; ^11^ Linha de Investigação Clínica e Interdisciplinar em Meio Ambiente, Genética e Oncobiologia (CIMAGO), Faculdade de Medicina da Universidade de Coimbra, Coimbra, Portugal; ^12^ Real-World Evidence & Late Phase, CTI Clinical Trial & Consulting Services Unipessoal Lda, Lisboa, Portugal

**Keywords:** systemic lupus erythematosus, antiphospholipid syndrome, biological therapies, small molecules, systemic autoimmune diseases, recommendations, Sjögren’s syndrome

## Abstract

Systemic lupus erythematosus (SLE), antiphospholipid syndrome (APS), and Sjögren’s syndrome (SS) are heterogeneous autoimmune diseases. Severe manifestations and refractory/intolerance to conventional immunosuppressants demand other options, namely biological drugs, and small molecules. We aimed to define evidence and practice-based guidance for the off-label use of biologics in SLE, APS, and SS. Recommendations were made by an independent expert panel, following a comprehensive literature review and two consensus rounds. The panel included 17 internal medicine experts with recognized practice in autoimmune disease management. The literature review was systematic from 2014 until 2019 and later updated by cross-reference checking and experts’ input until 2021. Preliminary recommendations were drafted by working groups for each disease. A revision meeting with all experts anticipated the consensus meeting held in June 2021. All experts voted (agree, disagree, neither agree nor disagree) during two rounds, and recommendations with at least 75% agreement were approved. A total of 32 final recommendations (20 for SLE treatment, 5 for APS, and 7 for SS) were approved by the experts. These recommendations consider organ involvement, manifestations, severity, and response to previous treatments. In these three autoimmune diseases, most recommendations refer to rituximab, which aligns with the higher number of studies and clinical experience with this biological agent. Belimumab sequential treatment after rituximab may also be used in severe cases of SLE and SS. Second-line therapy with baricitinib, bortezomib, eculizumab, secukinumab, or tocilizumab can be considered in SLE-specific manifestations. These evidence and practice-based recommendations may support treatment decision and, ultimately, improve the outcome of patients living with SLE, APS, or SS.

## 1 Introduction

Systemic lupus erythematosus (SLE), antiphospholipid syndrome (APS), and Sjögren’s syndrome (SS) are systemic autoimmune diseases (SAIDs) characterized by changes in immunity and inflammation pathways (1). As a result, several body tissues may be affected by the patient’s immune system. SAIDs often present relapse-remission courses, and critical flares or severe manifestations can occur, sometimes life-threatening (2).

The Standard of care (SoC) in SAIDs consists of treatment with corticosteroids and conventional immunosuppressive drugs (3). These agents are effective in most patients, but side effects and refractory cases can occur (3, 4). For that reason, drugs with a better benefit/risk profile are needed, especially after inadequate control by SoC (5). Biologic agents, immunoglobulins, and biotechnology small molecules have become a hallmark in the treatment of severe manifestations of SAIDs, alone or as adjunctive therapy to conventional immunosuppressive drugs (3, 6). However, many of these drugs have been used off-label due to a lack of randomized trials in such heterogenous and often rare conditions. Furthermore, despite its efficacy and often safe profile, biological therapy has frequently higher costs and access constraints (3, 4).

Within this context, the Study Group of Autoimmune Diseases of the Portuguese Society of Internal Medicine (NEDAI) aimed to review the evidence (from clinical trials and real-world settings) and to define recommendations for the off-label use of biologics in SAIDs, namely, SLE, APS, and SS.

## 2 Methods

**Figure 1:**
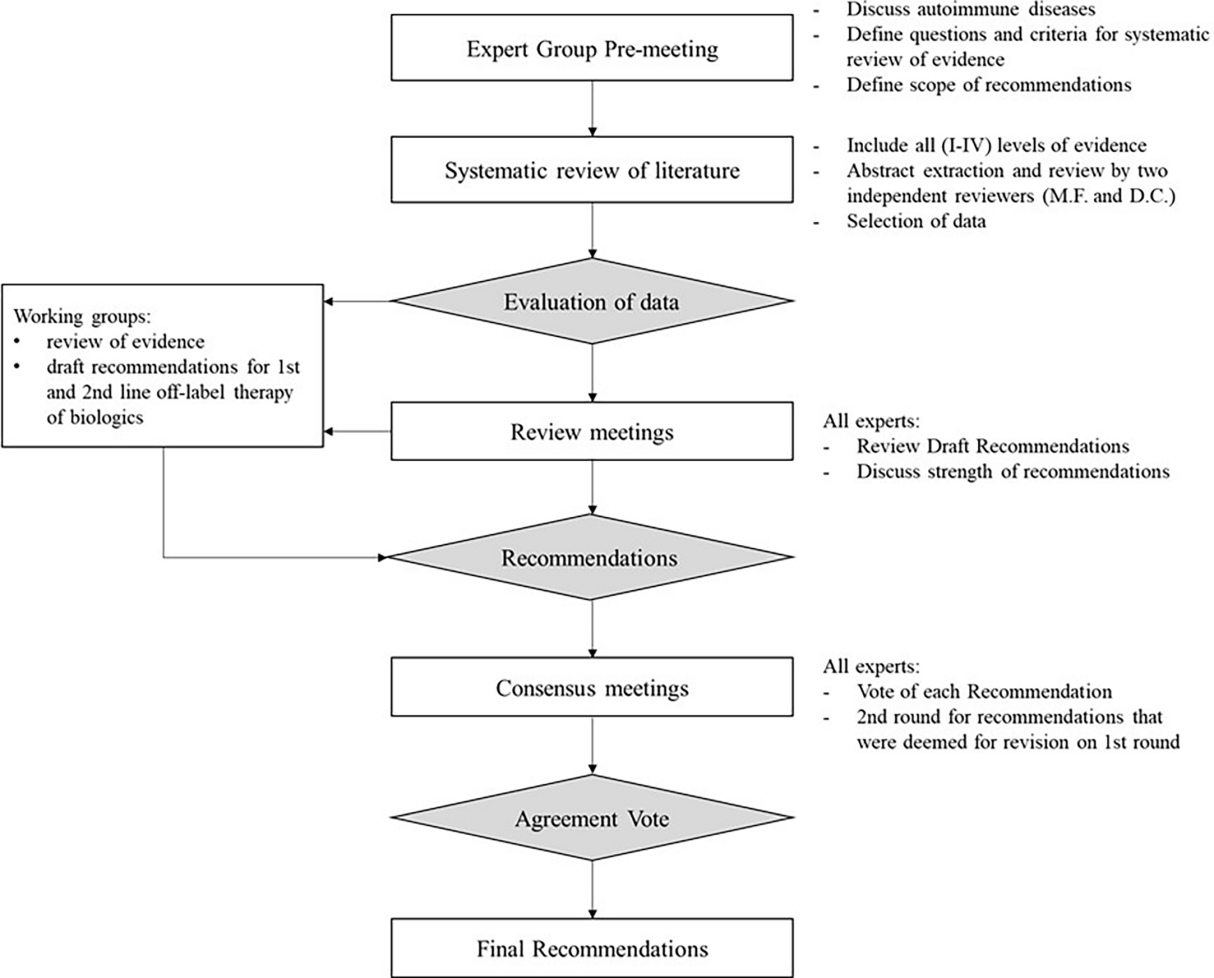
Definition and approval of recommendations – flowchart.

Recommendations were made by an independent expert panel, following a comprehensive literature review and two consensus rounds (**Figure 1**). This is a useful methodology when RCT evidence is limited and when the clinical questions to be addressed are clearly defined (2, 7).

All NEDAI members in 2019 (n=19) were invited and 17 experts participated. All experts have a long-standing experience in the care of SAIDs patients at university and tertiary hospitals in Portugal.

Two preliminary expert meetings were conducted in June 2018 and June 2019, to define the scope of recommendations. The following clinical questions and definitions were considered:

- Can “biologic therapy” be considered as 1^st^/2^nd^ line treatment? In which clinical circumstances? Which “biologic drug” can be used in 1^st^/2^nd^ line, considering the affected organ/clinical setting?
- “Biologic therapy” included all biological agents, small molecules, and immunoglobulins.
- Off-label use: the use of biological therapy in unapproved SAIDs (in Europe), regardless of being approved for the treatment of other diseases.
- 1^st^ line therapy: biologic therapy used concomitantly to or after glucocorticoid use.
- 2^nd^ line therapy: biologic therapy used after SoC immunosuppressive drugs or 1^st^ line biological agents.

A systematic review was conducted on PubMed. The inclusion criteria were defined before the literature search. Publications that met all the following criteria were included: 1) evaluation of treatment with biologic agent/small molecule; 2) in one of the defined SAIDs, 3) among adult patients, 4) irrespective of the type of study (case reports and reviews were included), and 5) written in English. Publications reporting diseases other than the defined ones or secondary SAIDs, diagnosis or identification of risk factors, molecular or biomarker evaluation, or *in vitro* or *ex vivo* studies, were excluded. Screening of publications was conducted by two independent reviewers. Cases of non-agreement were debated and, when required, a third reviewer was consulted. The process and results from the systematic review are presented in **Supplementary Material**.

Working groups, with experts allocated to only one disease, were provided with an overview of the resulting evidence to produce a set of preliminary recommendations for biologic as 1^st^ and 2^nd^ therapy line. The writing of recommendations was overviewed by all experts during a meeting in February 2020. After the revisions of the working groups, recommendations were classified according to the level of evidence, and the strength of recommendation, according to the 2016 standards of the Oxford Centre for Evidence-Based Medicine (**Supplementary Material**) (8). Experts also defined six categories for clinical use, based on the available evidence and their clinical experience (**Table 1**).

**Table 1:** Guidance for off-label use of biological therapy.

	Categories	Availability
I	Recommended use	Often used in clinical practice for other autoimmune diseases
II	Suggested use	Used in patients with specific characteristics or underlying clinical conditions
III	Possible use	Not contraindicated and successful case reports/series available
IV	Use not recommended	No additional benefits
V	Use not recommended	Contraindicated use

Draft recommendations were then voted during a remote consensus meeting in June 2021 (**Supplementary Material**). If the degree of agreement (i.e., the number of experts who agreed with the recommendation over the total number of experts) was lower than 75%, the recommendation was revised by the working group and, if applicable, submitted to a second consensus round (online survey) in September 2021. **Tables 2**–**4** present only the approved recommendations (i.e., ≥75% agreement).

**Table 2:** Recommendations for off-label biologic therapy of systemic lupus erythematosus.

Recommendation (clinical circumstance and drug)	LoE ^a)^	GoR ^a)^	Off-label guidance ^b)^
First-line therapy			
1. In pts with very active disease (i.e., SLEDAI>20 or BILAG 3A’s)			
RTX is recommended	2a	B	I
RTX-BEL may be used	2b	B	II
2. In pts with severe hemolytic anemia or severe thrombocytopenia (i.e., risk of death or organ damage)			
RTX is recommended	4	C	I
3. In pts with severe kidney disease (stage IV, presence of glomerular crescents and/or renal failure [GFR < 60 ml/min/1.73 m^2^])			
RTX is recommended	2a	C	I
RTX-BEL may be used	2b	C	II
4. In pts with severe CNS disease			
RTX is recommended	4	C	I
RTX-BEL may be used in recurrent cases	4	C	II
Second-line therapy			
5. In pts with persistently active disease for at least one year, with flares			
RTX is recommended in RTX-naïve cases	2a	C	I
baricitinib may be used in pts with predominant arthritis flares	1b	B	II
tocilizumab can be considered in pts with predominant arthritis flares	4	C	III
6. In pts with severe kidney disease,			
RTX is recommended in RTX-naïve cases	2a	C	I
RTX-BEL may be used in multi-refractory cases	4	C	II
secukinumab can be considered in multi-refractory cases	4	D	III
7. In pts with very active disease,			
RTX is recommended in RTX-naïve cases	2a	B	I
RTX-BEL may be used in RTX-naïve cases	2b	B	I
bortezomib can be considered in multi‐refractory patients	4	D	III
8. In pts with hemolytic anemia or thrombocytopenia,			
RTX is recommended in RTX-naïve cases	4	C	I
bortezomib can be considered in multi‐refractory cases	4	D	III
9. In pts with moderate or severe CNS disease,			
RTX is recommended in RTX-naïve cases	4	C	I
RTX-BEL may be used in multi-refractory cases	4	D	II

LoE, Level of Evidence; GoR, Grade of Recommendation; BILAG, British Isles Lupus Assessment Group index; CNS, Central Nervous System; GFR, Glomerular Filtration Rate; pts, patients; RTX, rituximab; RTX-BEL, rituximab, and belimumab (sequential therapy); SLEDAI, Systemic Lupus Erythematosus Disease Activity Index.

a LoE and GoR according to the Oxford CEMBE 2009 guidelines (see **Table S1**).

b Guidance of off-label use of biologic therapy, as defined by experts (see **Table 1**).

**Table 3:** Recommendations for off-label biologic therapy of antiphospholipid syndrome.

Recommendation (clinical circumstance and drug)	LoE ^a)^	GoR ^a)^	Off-label guidance ^b)^
First-line therapy			
1. RTX is recommended as first-line therapy for APS patients with severe thrombocytopenia	2b	B	I
2. The SoC in CAPS consists of combined therapy with anticoagulants, corticosteroids, plasmapheresis or IVIG, and systemic antibiotics if adequate	2b	D	I
3. In patients with CAPS, RTX may be added to the combined therapy	2b	D	II
4. In patients with CAPS and other SAIDs (e.g., SLE), RTX may be added to combined therapy	4	D	II
Second-line therapy			
5. As second-line therapy, the evidence available is insufficient to support any recommendations	–	–	–

LoE, Level of Evidence; GoR, Grade of Recommendation; APS, antiphospholipid syndrome; CAPS, catastrophic antiphospholipid syndrome; IVIG, intravenous immunoglobulin; RTX, rituximab; SAIDs, systemic autoimmune diseases; SLE, systemic lupus erythematosus; SoC, standard of care.

^a^LoE and GoR according to the Oxford CEMBE 2009 guidelines (see **Table S1**).

^b^Guidance of off-label use of biologic therapy, as defined by experts (see **Table 1**).

**Table 4:** Recommendations for the off-label biological therapy in Sjögren’s syndrome.

Recommendation (clinical circumstance and drug)	LoE ^a)^	GoR ^a)^	Off-label guidance ^b)^
First-line therapy			
1. In pts with SS and with sicca symptoms only, biological therapy is not recommended.	1a	A	IV
2. In pts with SS and severe systemic manifestation, with risk of lymphoma (at least 3 risk factors for lymphoma) and recent onset (<12 months of evolution), RTX can be used as first-line therapy.	4	C	II
3. RTX may be used as first-line therapy in pts with SS (<12 months of evolution) and peripheral neuropathy, severe thrombocytopenia, severe CNS disease, severe parotid swelling, and/or cryoglobulinemic vasculitis.	1b	B	II
4. RTX may be used as first-line therapy in pts with SS (>12 months of evolution) and peripheral neuropathy, severe thrombocytopenia, severe CNS disease, severe parotid swelling, and/or cryoglobulinemic vasculitis.	2b	C	II
5. Fatigue in SS patients is not a recommendation for biological therapy.	1a	A	IV
Second-line therapy			
6. RTX is recommended in pts with refractory SS who present the systemic manifestations indicated for the first-line therapy.	1b	B	I
7. In RTX-experienced pts, sequential therapy of BEL-RTX may be used.	2b	C	II

LoE, Level of Evidence; GoR, Grade of Recommendation; BEL, belimumab; CNS, central nervous system; SS, Sjögren’s syndrome; pts, patients; RTX, rituximab.

^a^LoE and GoR according to the Oxford CEMBE 2009 guidelines (see **Table S1**).

^b^Guidance of off-label use of biologic therapy, as defined by experts (see **Table 1**).

## 3 Recommendations

### 3.1 Off-label biologic therapy in systemic lupus erythematosus

#### 3.1.1 Disease background

SLE can affect several organs, including the skin, kidney, heart, lungs, and the hematological, musculoskeletal, and nervous systems (2, 9). The incidence of flares is about 0.65 per patient-year and even though the 10-year survival is higher than 90% (10), about one-third of SLE deaths seem to result from disease activity (11). Hence, treatment should aim at preventing damage and maintaining disease control whilst using the lowest possible dose of corticosteroids, ultimately aiming at reducing morbidity and mortality (2, 9, 12). However, up to 20% of SLE patients do not adequately respond to conventional treatments with corticosteroids and immunosuppressive drugs (10). In addition, drug-induced toxicity is frequent, particularly in patients receiving longer treatments and those with refractory disease and/or lupus nephritis (LN) (13).

Data about most biological therapies in SLE treatment is scattered, although several RCTs were conducted and RTX has been extensively used (14). Belimumab is approved for SLE treatment, as add-on therapy in adult patients with high disease activity despite SoC therapy and in combination with background immunosuppressive therapies for the treatment of adult patients with active LN. A pooled analysis of belimumab RCTs confirmed its efficacy irrespective of concomitant medication (15), with an improvement in disease activity, particularly in the musculoskeletal and mucocutaneous organ domains (16), a better quality of life (17), reduction of long-term organ damage (18) and effectiveness maintained for up to 7 and 12 years (19). A two-year phase III RCT showed an improvement in renal response among active LN patients that received belimumab plus standard therapy while reducing the risk of renal-related events or death (20). The efficacy of belimumab was also observed in several open-label and real-world studies (21).

*Anifrolumab* is a type I interferon receptor antagonist that reduces disease activity in patients with moderate-to-severe SLE (22, 23). A phase III RCT (TULIP 2 study) demonstrated anifrolumab efficacy through a composite endpoint at week 52 – reduction in any moderate-to-severe baseline disease activity and no worsening in any of nine BILAG organ systems, no SLEDAI worsening, no increase of ≥0.3 points in the Physician Global Assessment of disease activity (24). Anifrolumab was authorized by EMA in 2022, as an add-on therapy for the treatment of adult patients with moderate to severe, active autoantibody-positive SLE, despite standard therapy.

#### 3.1.2 Definitions

- SLE with inadequate response to SoC: residual disease activity not allowing tapering of glucocorticoids to less than 5 mg/day and/or frequent relapses (2).
- very active SLE: patients scoring SLEDAI >20 or BILAG 3A’s (25).
- persistent active SLE: patients presenting some disease activity for 1 or more years (26).
- flare: measurable increase in disease activity usually leading to a change of treatment (2).
- severe kidney disease: defined as stage IV nephritis, presence of glomerular crescents, and/or renal failure (GFR < 60 ml/min/1.73 m^2^).
- severe CNS disease: defined as neuropsychiatric or ophthalmological involvement.

#### 3.1.3 Recommendations and summary of evidence

Recommendations for the off-label biological therapy of SLE are presented in **Table 2**.

##### 3.1.3.1 Rituximab

*RTX is recommended as 1^st^ line therapy in patients with very active disease, severe hemolytic anemia, or thrombocytopenia with risk of death or organ damage, severe kidney disease (Stage 4, presence of glomerular crescents and/or renal failure), or with severe CNS disease. RTX is recommended as 2^nd^ line therapy in patients with persistently active disease for at least one year with flares and particularly in patients with very active disease, hemolytic anemia or thrombocytopenia, severe kidney disease, or moderate or severe CNS disease.*

Even though RCTs had not shown the efficacy of RTX in the treatment of SLE (27) and LN (28), several observational studies – including registries (29–32) and large retrospective studies (33–35) – showed RTX effectiveness and a low incidence of adverse events in the treatment of refractory SLE, including in patients more treatment-experienced (36). These differences between observational studies and RCTs may be due to the heterogeneity of the SLE population in the real-world setting and some methodological problems of RCTs (such as lack of power and selection of endpoints) (37, 38). Some meta-analyses that included observational results demonstrated the RTX effectiveness in SLE and LN, with complete response estimates of 46%-57% and 36% 51%, respectively (38–40).

The successful use of RTX as induction therapy in refractory LN was also reported in several studies (41), including for patients with poorer prognostic factors in terms of renal disease (42) and patients with membranous LN (type V) (43). A pooled analysis of data from European cohorts showed that add-on treatment with RTX was successful in patients with type IV and type V LN – although less than in patients with type III (44). A recent network meta-analysis placed add-on therapy with RTX in line with combination treatment with leflunomide and tacrolimus but, since the population treated with RTX seems to have more severe disease, conclusions may have been biased (45).

RTX was used successfully in the treatment of SLE-associated refractory cytopenia (46, 47), including thrombocytopenia (48) and autoimmune hemolytic anemia (AIHA) (49). Additionally, a meta-analysis demonstrates the effectiveness of RTX in the treatment of AIHA and microangiopathic hemolytic anemia (50). A case report indicated the effectiveness of RTX treatment in corticosteroid-resistant immune thrombocytopenia purpura associated with SLE (51).

The effectiveness of RTX treatment was also reported in refractory cases with neuropsychiatric involvement, in patients with delirium or psychosis (52–54), and in the presence of ophthalmologic manifestations (55). Furthermore, some case reports suggest that RTX may be useful as 1^st^ line therapy in severe neuropsychiatric SLE (56), including demyelinating syndrome secondary to SLE, severe cognitive dysfunction, brainstem disease, cranial nerve palsies, and weakness and numbness in limbs (57) or with concurrent neuropsychiatric and renal involvement (58).

##### 3.1.3.2 Sequential therapy with Rituximab followed by Belimumab

*The sequential therapy of RTX followed by belimumab may be used as 1^st^ line therapy in patients with very active disease or with severe kidney disease (Stage IV, presence of glomerular crescents and/or renal failure [GFR < 60 ml/min/1.73 m^2^]) and can be considered in patients with severe CNS disease. RTX-belimumab is recommended as 2^nd^ line therapy in RTX-experienced patients with very active disease, severe kidney disease, or moderate to severe CNS disease.*

While the efficacy of belimumab has been demonstrated in SLE, some patients maintain disease activity. In line with several case reports (59), the phase II SynBioSe study showed significant clinical and immunological improvements from baseline in patients with severe refractory SLE who received RTX followed by belimumab, and that patients maintained response over two years (60, 61). Other studies described the successful treatment of LN refractory to RTX (62, 63), although the CALIBRATE study (an open-label phase II RCT) showed no clinical improvement of belimumab infusions after RTX plus cyclophosphamide, for treatment of refractory LN (64). One case was reported with a successful result of sequential treatment in neuropsychiatric SLE (65). The BEAT-LUPUS study (66) and the BLISS-BELIEVE study (67) evaluated this combination, and findings suggest that adding a single cycle of RTX to belimumab therapy does not improve disease control rates. However, patients with anti–double–stranded (ds)DNA antibodies showed a significantly greater decrease in median levels at 52 weeks among those in the belimumab-RTX group than those in the belimumab-placebo arm, with reductions of 69.2% versus 46.1% (68).

##### 3.1.3.3 Baricitinib

*Baricitinib may be used as 2^nd^ line therapy in patients with persistently active disease and predominant arthritis flares.* Baricitinib is an oral selective Janus kinase (JAK)1 and JAK2 inhibitor approved for the treatment of rheumatoid arthritis and atopic dermatitis (69). A double-blind phase II RCT, with 314 active SLE patients involving skin or joints showed that baricitinib 4mg significantly improved the signs and symptoms of active SLE – with a greater proportion (vs. placebo) of patients achieving resolution of arthritis/rash – in patients who failed adequate control with an SoC approach (70). A high rate of serious infections was observed, although similar to what was observed with belimumab (70–72). On January 2022, the phase 3 development program of baricitinib in SLE treatment was stopped due to poor efficacy results in the SLE-BRAVE-II trial (NCT03616964), even though the SLE-BRAVE-I (NCT03616912) showed a significant reduction in disease activity as evaluated by the SRI-4 standard measurement tool.

##### 3.1.3.4 Bortezomib

*In patients with very active disease and RTX-experienced, bortezomib can be considered as 2^nd^ line therapy.* Bortezomib reduced disease activity in refractory SLE (73) and LN (74, 75) and treated successfully one SLE patient with warm-type hemolytic anemia refractory to RTX (76). However, its safety profile requires the monitoring of adverse events, such as peripheral neuropathy and hypogammaglobulinemia (77). A double-blind RCT failed to demonstrate bortezomib efficacy after a high discontinuation rate occurred, due to adverse events (78). A case report showed that lower doses of bortezomib (i.e., with longer intervals for the administration of the drug) could be useful for the treatment of patients with concomitant multiple myeloma, although the patient presented mildly active SLE (77). Sequential treatment with belimumab may reduce the regeneration of autoreactive B cells, according to a report of two cases (79).

##### 3.1.3.5 Eculizumab

*In patients with refractory lupus nephritis, eculizumab can be considered in multi-refractory cases.* Eculizumab is a recombinant humanized monoclonal antibody (mAb) that binds to the complement component C5 and prevents its activation (80). A placebo-controlled, double-blind phase I RCT with 24 SLE patients failed to demonstrate the efficacy of eculizumab, according to laboratory and clinical parameters and SLEDAI scores (81). However, case reports described good results with eculizumab in the treatment of refractory LN (82, 83). In a review of SLE with renal involvement, irrespective of concomitant LN, all patients (n=6) showed a sustained improvement in renal function and normalization of complement parameters after treatment with eculizumab (median follow-up of 9 months) (80). This successful response was also observed in patients with refractory thrombotic microangiopathy associated with LN or SLE (84).

##### 3.1.3.6 Secukinumab

*In patients with active lupus nephritis, secukinumab can be considered as 2^nd^ line therapy in multi-refractory cases.* Secukinumab is a human IgG1κ mAb that binds to the interleukin (IL)-17A. It has been suggested for the treatment of lupus since T-helper 17 cells are involved in the SLE pathogenesis (85). A case report of a 62-year-old female who presented with psoriasis vulgaris and refractory LN – showing proliferation of activated T helper 17 cells in peripheral blood, and renal infiltration of IL-17-positive lymphocytes – was treated successfully with secukinumab, for both psoriasis and LN (86). An ongoing phase 3 RCT will evaluate subcutaneous secukinumab *vs.* placebo, in combination with SoC, in patients with active LN (NCT04181762).

##### 3.1.3.7 Tocilizumab

*Tocilizumab can be considered in persistent active SLE for at least one year with predominant arthritis flares.* An open-label, phase I study with 16 patients with mildly to moderately active SLE showed clinical and serological response after tocilizumab treatment (87). Its use as add-on therapy in SLE has been described in case reports, with successful outcomes in patients with arthritis flares (88, 89) or with refractory serositis (90, 91). Tocilizumab was also used successfully in one SLE patient with AIHA refractory to RTX treatment (92). Neutropenia and the increased risk of infection limit tocilizumab use in SLE treatment (87).

##### 3.1.3.8 Other biologics

RCTs have failed to demonstrate *abatacept* efficacy in the treatment of active LN (93, 94) or SLE (95), although some exploratory endpoints related to articular involvement showed good results.

A phase I RCT showed that 5 out of 12 patients treated with *dapirolizumab* achieved an SRI-4 response by week 12 (vs 1 out of 7 in the placebo group) (96). However, the phase IIb RCT in adults with moderately-to-severely active SLE failed to meet its primary endpoint at week 24, despite the improvement of other secondary endpoints and biomarkers (97). A phase III study is ongoing (NCT04294667).

*Daratumumab*, a monoclonal antibody targeting CD38, induced substantial clinical responses in cases with life-threatening lupus, sustained afterward by maintenance therapy with belimumab (98, 99).

According to some reviews, *IVIG* can be considered in acute severe flares or refractory SLE, as well as in LN treatment (100, 101). IVIG was also used successfully in the treatment of SLE-associated severe myelitis (102).

A prospective, open-label, single-arm, phase I/IIa trial evaluated the safety, tolerability, and response of Treg to low-dose *interleukin-2* (IL-2) in patients with active and refractory SLE (103). Even though the responsiveness to IL-2 in Treg from SLE patients showed no impairment, the clinical response was transient and declined almost to baseline levels in between the cycles, suggesting that the cyclic treatment modality may be suboptimal.

The use of anti-tumor necrosis factor (TNFα) in SLE is controversial, due to the risk of disease flare (14, 104). The short-term use of *infliximab* was successful in an open-label study with moderately active SLE patients (105). However, patients with lupus arthritis (n=5) maintained clinical response for less than 2 months after the last infusion. Long-term therapy was also associated with serious adverse events (SAEs) in two patients.

*Obinutuzumab* combined with mycophenolate and steroids was evaluated in active III/IV LN patients in a phase II, placebo-controlled RCT. At week 52, a higher proportion of obinutuzumab-treated patients achieved response (though not statistically significant), and a statistically significant improvement of 19% was observed at week 104 regarding complete renal response (106). In a small observational study with 4 non-responders to RTX who switched to *ocrelizumab*, three achieved and maintained clinical response during the following 5 years but six SAEs were observed (four serious infections) (107). Regarding *ofatumumab*, one case series with SLE patients intolerant to RTX showed that in 12 patients with LN, half achieved renal remission after 6 months of ofatumumab treatment (108).

Anti-CD19 *CAR T cell therapy* was evaluated in five patients with refractory SLE, who achieved SLE remission after 3 months following a well-tolerated treatment (109).

### 3.2 Off-label biologic therapy in antiphospholipid syndrome

#### 3.2.1 Disease background

APS is characterized by the presence of persistent antiphospholipid antibodies (aPL) leading to thrombosis in veins, arteries, and microvasculature as well as obstetrical complications (110). About 1% of patients develop catastrophic APS (CAPS), a severe and frequently fatal manifestation (111, 112). CAPS is defined as small vessel thrombosis in three or more organs, systems, and/or tissues either simultaneously or within 1 week, with histological confirmation of small vessel occlusion, in the presence of persistent aPL and absence of vasculitis (113). Patients with other SAIDs (most frequently SLE) can also present aPL, increasing the risk of thrombotic events (110).

Thrombosis prevention requires anticoagulation and anti-platelet aggregating agents (114). For patients with recurrent thrombosis, fluctuating INR, or for those who are at high risk of major bleeding, alternative therapies may be considered, including low-molecular-weight heparin, hydroxychloroquine, or statins (110). For patients with CAPS, acute management is based on a combined therapy using anticoagulation, corticosteroids, plasma exchange, and/or intravenous immunoglobulin administration and, in the case of CAPS initiated by an infectious event, systemic antibiotics (110, 113–115). Some recommendations suggest the use of rituximab for refractory APS patients (116), and rituximab or eculizumab for refractory CAPS (113, 114, 117).

#### 3.2.2 Recommendations and summary of evidence

Recommendations for the off-label biological therapy of APS and CAPS are presented in **Table 3**.

##### 3.2.2.1 Intravenous immunoglobulin

*Acute treatment with anticoagulants, corticosteroids, and IVIG is the current SoC of CAPS.* The CAPS registry has shown significantly lower mortality among CAPS patients receiving combination therapy, compared with those receiving other treatments (odds ratio [OR], 0.51; 95% confidence interval [CI], 0.27, 0.95) (117).

Even though the paucity and equivocal evidence retrieved from publications between 2014 and 2019, IVIG has been used in the treatment of APS patients with recurrent thrombosis (118), obstetric APS (119–122), and CAPS (123). Usually administered at doses of 0.4 g/kg/day for 5 days, IVIG may be more useful in patients with thrombocytopenia and has the advantage of being immunomodulatory rather than immunosuppressive (117, 118). However, IVIG was also associated with both increased thrombotic risk and worsening renal function, especially in elderly patients (117).

##### 3.2.2.2 Rituximab

*RTX may be used as 1^st^ line therapy in CAPS patients, added to combination therapy (glucocorticoids, anticoagulation, plasmapheresis, and/or IVIG and systemic antibiotics if adequate).* RTX has been reported to be successful in the acute treatment of CAPS first episodes, as part of combination treatment (124, 125), especially in the case of life-threatening complications (126). The CAPS registry showed that, among 20 patients treated with RTX at 375 mg/m^2^ weekly for 4 weeks or 1g every 14 days for 2 sessions, 75% (n=15) recovered from whom 87% (n=13) had no recurrent thrombosis during follow-up (115, 127). Lack of response was reported in one patient with a subacute recurrence of CAPS treated with combination therapy and rituximab (128), and in one patient with diffuse alveolar hemorrhage treated with RTX and glucocorticoids only (129).

*Patients with CAPS and other SAIDs, namely SLE, may benefit from RTX added to combination therapy (glucocorticoids, anticoagulation, IVIG, systemic antibiotics if adequate).* A single-center retrospective analysis showed consistent improvement in 5 out of 6 patients with SLE-associated APS after treatment with rituximab (130). Indications for RTX therapy were a failure in warfarin therapy despite the adequate target INR (4 cases), and life-threatening active disease refractory to conventional therapy (one case of transverse myelitis, and another of diffuse alveolar hemorrhage). All patients received prior conventional therapy. RTX also showed efficacy in case reports of SLE-associated APS (131) or CAPS (132, 133).

*RTX can be used as 1^st^ line therapy in APS patients with severe thrombocytopenia.* An open-label pilot study with 19 patients aPL positive reported that RTX had some efficacy in controlling manifestations such as thrombocytopenia, hemolytic anemia, and skin ulcers (134).

##### 3.2.2.3 Other biologic therapy

*Eculizumab* has been reported to successfully treat patients with refractory CAPS, and patients with renal transplants and APS or CAPS, and some authors suggest its use in refractory patients who are refractory to other therapeutics (135, 136).

### 3.3 Off-label biologic therapy in Sjögren’s syndrome

#### 3.3.1 Disease background

Sjögren’s syndrome (SS) is among the most prevalent SAIDs and concurs frequently with other conditions, such as rheumatoid arthritis, SLE, scleroderma, or hypothyroidism, while SS (SS) shows a prevalence in Europe between 0.1% and 4.8% (137, 138). It is characterized by lymphocytic infiltration of the epithelium of exocrine glands, resulting in xerostomia and xerophthalmia (i.e., sicca symptoms) (139). Extra glandular involvement may occur in at least one-third of patients, with chronic fatigue, arthralgia, and organ involvement such as lungs, skin, kidneys, and nervous system (140).

Treatment of SS is mainly empirical and symptom-targeted (140, 141). Some recommendations suggest that the use of systemic immunosuppressive therapies, including glucocorticoids and immunoglobulins, should be restricted to patients with active systemic disease and only after evaluating the severity and organ damage (139, 142, 143).

#### 3.3.2 Definitions

- Overall severity of SS should be evaluated based on the EULAR Sjögren’s syndrome disease activity index (ESSDAI) (143).
- Severe systemic SS: patients with an ESSDAI score >14, or high activity in any of the ESSDAI domains (e.g., with organ damage) (143).
- Organ damage should consider blood (anemia, leukopenia, thrombocytopenia, lymphoma); kidney (diabetes insipidus, interstitial nephritis, glomerular disease); gastrointestinal (xerostomia, esophagitis, gastritis, primary biliary cholangitis), lung (interstitial pneumonitis); cardiovascular (vasculitis), serious swelling of the parotid gland, inflammatory arthritis, CNS involvement or peripheral neuropathy, and audition and visual disturbances.
- Refractory SS: patients for whom conventional therapies, including topical moisturizers, secretagogues, anti-inflammatories, and immunomodulators have proven to be insufficient.
- SS associated with other SAIDs should be treated according to these recommendations, in addition to the treatment of the associated SAID.

#### 3.3.3 Recommendations and summary of evidence

Recommendations for the off-label biological therapy in SS are presented in **Table 4**.

##### 3.3.3.1 Rituximab

*In SS patients with sicca symptoms only, the use of biological therapy is not recommended.* In the TEARS study (144), RTX did not show efficacy in reducing symptoms or disease activity in patients with SS at week 24 but did improve fatigue at weeks 6 and 16 of treatment, and the physician evaluation of disease activity at week 6. The TRACTISS study also failed to prove RTX efficacy in this population (145). Two meta-analyses reported the lack of RTX efficacy in SS, failing to improve lacrimal gland function, oral dryness, or fatigue at 6 months (146, 147).

*RTX may be used as 1^st^ line therapy in patients with SS and severe systemic manifestation, with risk of lymphoma (at least 3 risk factors for lymphoma) and recent onset (i.e., less than 12 months of evolution).* The Spanish GEAS-SS Registry (148) of SS patients with lymphoma treated with RTX-based chemotherapy regimens showed that 41 out of 64 patients achieved a complete response. In a retrospective study with SS patients with mucosa-associated lymphoid tissue-type lymphoma of the parotid gland, a complete response was observed in 5 out of 13 patients treated with RTX only and in all 6 patients with RTX-based chemotherapy (149).

*RTX may be used as 1^st^ line therapy in patients with SS (<12 months of evolution) and peripheral neuropathy, severe thrombocytopenia, severe CNS disease, severe parotid swelling, and/or cryoglobulinemic vasculitis.* One of the first RCTs with RTX (n=30 patients) described a significant reduction in reported extra-glandular manifestations and an improvement of the musculoskeletal features at weeks 12 and 36 (p=0.029) and vasculitis at week 24 (p=0.03) (150). Another RCT in patients with cryoglobulinemia (associated or not with SS) also had positive results (151). In a prospective cohort of 78 patients with high disease activity, RTX showed a good safety profile and was effective for the treatment of SS with systemic manifestation, based on the reduction of the ESSDAI and of daily dose of corticosteroids (152). In a series of 16 patients (153), RTX was associated with an improvement of the systemic manifestations in ≥80% of cases. Most studies included patients with a recent onset of SS, hence the lower strength for the recommendation that *RTX may be used as 1^st^ line therapy in patients with SS (>12 months of evolution) and multiple neuropathies, severe thrombocytopenia, severe CNS disease, severe parotid swelling and/or cryoglobulinemic vasculitis.*

*RTX is recommended as 2^nd^ line therapy in RTX-naïve patients with refractory SS and presenting lymphoma, multiple neuropathies, severe thrombocytopenia, severe CNS disease, severe parotid swelling, and/or cryoglobulinemic vasculitis.* A multicenter registry showed that, among 15 refractory SS patients treated with RTX due to extra-glandular involvement, 10 (67%) had a complete response, 3 (20%) a partial response, and 2 (13%) were non-responders after 12 months (154). A retrospective study showed the effect of RTX in improving SS patients with thrombocytopenia refractory to conventional immunosuppressive drugs (46). More recently, a single-center retrospective study reported that, among 10 female patients with severe or refractory SS involvement, six became asymptomatic, two had symptomatic improvement and two had no benefit, suggesting that RTX can be considered in these cases (155).

##### 3.3.3.2 Sequential therapy of Belimumab followed by Rituximab

*In RTX-experienced patients, sequential therapy of belimumab-RTX may be used.* The BELISS open-label trial showed a significant decrease in the mean ESSDAI score from 8.7 to 5.7 at week 28 (156). The primary composite endpoint was achieved in 18 (60%) patients – i.e., improvement in two of five items at week 28, comprising a 30% reduction in VAS scores of dryness, fatigue, pain, or physician-assessed systemic activity and/or >25% improvement in any B cell activation biomarker. Belimumab was effective in 3 out of 5 patients refractory to RTX. The BELISS extension showed that the improvement was significantly maintained in 19 patients that completed one year of treatment (157).

##### 3.3.3.3 Other biologic therapy

*Abatacept* showed good results in open-label studies, with a significant reduction of ESSDAI and an improvement in fatigue and quality of life (158), but failed phase III trials in the treatment of active SS (159, 160).

## 4 Conclusions

To our knowledge, it is the first attempt to establish recommendations regarding the use of biologic therapies in SAIDs, namely in clinical settings with evidence gaps regarding the use of these therapies. After an extensive literature review and consensus methodology by a large expert panel, a total of 32 recommendations were defined for SLE, APS, and SS. The higher number of recommendations for SLE treatment reflects the fact of being a less organ-specific syndrome, with pleomorphisms and multiple pathogenic mechanisms. APS and SS are also challenging to manage, especially when presenting with multisystemic and severe manifestations that can urge the prevention of extensive organ damage or even death, as happens in CAPS.

We acknowledge that supporting evidence is mostly derived from small trials and observational studies, except for some important RCTs on SLE. The scarcity of RCT is well recognized among SAIDs, thus limiting the evidence level of the recommendations, and increasing its susceptibility to experts’ own experience. Nevertheless, bias was minimized by an extensive literature search and the number of experts who voted on the recommendations.

The management of patients living with SAIDs is demanding and goes beyond treatment recommendations (161). Future updates of this guidance should address dimensions such as vaccination strategies as less is known about the immunization of APS and SS patients and about SARS-CoV-2 vaccines in SAIDs receiving biologic therapy (162), although some studies have addressed the immunization of SLE patients (163, 164).

There are many innovative drugs available and being developed, but not all are effective across all SAIDs. The same clinical manifestation does not imply the same pathophysiology, and the active pathway at a given time may change within the same disease, thus making the demand for effective treatments even more challenging. In addition, the high costs of biologics and, consequently, the limitations in Europe on timely access to innovative drugs are barriers that need to be addressed. This evidence and practice-based guidance can help other clinicians in their therapeutic decisions and, ultimately, improve the outcome for patients living with these conditions.

## Author contributions

Substantial contributions to study conception and design: AM, MF, CV. Drafting the article: JDA, JF, RF, MF, CV. Review of the article for important intellectual content: All authors. All authors contributed to the article and approved the submitted version. 

## References

[1] Rose NR. Prediction and prevention of autoimmune disease in the 21st century: A review and preview. Am J Epidemiol (2016) 183(5):403–6. doi: 10.1093/aje/kwv292 26888748

[2] Fanouriakis A Kostopoulou M Alunno A Aringer M Bajema I Boletis JN. Update of the EULAR recommendations for the management of systemic lupus erythematosus. Ann Rheum Dis (2019) 78(6):736–45. doi: 10.1136/annrheumdis-2019-215089 30926722

[3] Carballido JM Regairaz C Rauld C Raad L Picard D Kammuller M. The emerging jamboree of transformative therapies for autoimmune diseases. Front Immunol (2020) 11:472. doi: 10.3389/fimmu.2020.00472 32296421 PMC7137386

[4] Fugger L Jensen LT Rossjohn J. Challenges, progress, and prospects of developing therapies to treat autoimmune diseases. Cell (2020) 181(1):63–80. doi: 10.1016/j.cell.2020.03.007 32243797

[5] Kaegi C Wuest B Schreiner J Steiner UC Vultaggio A Matucci A. Systematic review of safety and efficacy of rituximab in treating immune-mediated disorders. Front Immunol (2019) 10:1990. doi: 10.3389/fimmu.2019.01990 31555262 PMC6743223

[6] Rosman Z Shoenfeld Y Zandman-Goddard G. Biologic therapy for autoimmune diseases: An update. BMC Med (2013) 11:88. doi: 10.1186/1741-7015-11-88 23557513 PMC3616818

[7] Kleinmann JF Tubach F Le Guern V Mathian A Richez C Saadoun D. International and multidisciplinary expert recommendations for the use of biologics in systemic lupus erythematosus. Autoimmun Rev (2017) 16(6):650–7. doi: 10.1016/j.autrev.2017.04.011 28434948

[8] OCEBM Levels of Evidence Working Group. The Oxford 2011 Levels of Evidence. Oxford: Oxford Centre for Evidence-Based Medicine (2011). Available at: http://www.cebm.net/ocebm-levels-of-evidence/.

[9] Kaul A Gordon C Crow MK Touma Z Urowitz MB van Vollenhoven R. Systemic lupus erythematosus. Nat Rev Dis Primers (2016) 2:16039. doi: 10.1038/nrdp.2016.39 27306639

[10] Bertsias G Cervera R Boumpas DT European League Against Rheumatism. Systemic lupus erythematosus: Pathogenesis and clinical features. In: Bijlsma JWJ, editor. EULAR textbook on rheumatic diseases, vol. 5 . London: BMJ / EULAR (2012). p. 476–505.

[11] Doria A Iaccarino L Ghirardello A Zampieri S Arienti S Sarzi-Puttini P. Long-term prognosis and causes of death in systemic lupus erythematosus. Am J Med (2006) 119(8):700–6. doi: 10.1016/j.amjmed.2005.11.034 16887417

[12] van Vollenhoven RF Mosca M Bertsias G Isenberg D Kuhn A Lerstrom K. Treat-to-Target in systemic lupus erythematosus: Recommendations from an international task force. Ann Rheum Dis (2014) 73(6):958–67. doi: 10.1136/annrheumdis-2013-205139 24739325

[13] Samotij D Reich A. Biologics in the treatment of lupus erythematosus: A critical literature review. BioMed Res Int (2019) 2019:8142368. doi: 10.1155/2019/8142368 31396534 PMC6668536

[14] Magro R. Biological therapies and their clinical impact in the treatment of systemic lupus erythematosus. Ther Adv Musculoskelet Dis (2019) 11:1759720X19874309. doi: 10.1177/1759720X19874309 PMC6755633 31565077

[15] Schwarting A Dooley MA Roth DA Edwards L Thompson A Wilson B. Impact of concomitant medication use on belimumab efficacy and safety in patients with systemic lupus erythematosus. Lupus (2016) 25(14):1587–96. doi: 10.1177/0961203316655215 PMC5089223 27488472

[16] Manzi S Sanchez-Guerrero J Merrill JT Furie R Gladman D Navarra SV. Effects of belimumab, a b lymphocyte stimulator-specific inhibitor, on disease activity across multiple organ domains in patients with systemic lupus erythematosus: Combined results from two phase III trials. Ann Rheum Dis (2012) 71(11):1833–8. doi: 10.1136/annrheumdis-2011-200831 PMC3465857 22550315

[17] Strand V Levy RA Cervera R Petri MA Birch H Freimuth WW. Improvements in health-related quality of life with belimumab, a B-lymphocyte stimulator-specific inhibitor, in patients with autoantibody-positive systemic lupus erythematosus from the randomised controlled BLISS trials. Ann Rheum Dis (2014) 73(5):838–44. doi: 10.1136/annrheumdis-2012-202865 PMC3995218 23524886

[18] Bruce IN Urowitz M van Vollenhoven R Aranow C Fettiplace J Oldham M. Long-term organ damage accrual and safety in patients with SLE treated with belimumab plus standard of care. Lupus (2016) 25(7):699–709. doi: 10.1177/0961203315625119 26936891 PMC4958991

[19] Ginzler EM Wallace DJ Merrill JT Furie RA Stohl W Chatham WW. Disease control and safety of belimumab plus standard therapy over 7 years in patients with systemic lupus erythematosus. J Rheumatol (2014) 41(2):300–9. doi: 10.3899/jrheum.121368 24187095

[20] Furie R Rovin BH Houssiau F Malvar A Teng YKO Contreras G. Two-year, randomized, controlled trial of belimumab in lupus nephritis. N Engl J Med (2020) 383(12):1117–28. doi: 10.1056/NEJMoa2001180 32937045

[21] Trentin F Gatto M Zen M Larosa M Maddalena L Nalotto L. Effectiveness, tolerability, and safety of belimumab in patients with refractory SLE: A review of observational clinical-practice-based. Clin Rev Allergy Immunol (2018) 54(2):331–43. doi: 10.1007/s12016-018-8675-2 PMC6132773 29512034

[22] Morand EF Trasieva T Berglind A Illei GG Tummala R. Lupus low disease activity state (LLDAS) attainment discriminates responders in a systemic lupus erythematosus trial: Post-hoc analysis of the phase IIb MUSE trial of anifrolumab. Ann Rheum Dis (2018) 77(5):706–13. doi: 10.1136/annrheumdis-2017-212504 PMC5909750 29420200

[23] Furie R Khamashta M Merrill JT Werth VP Kalunian K Brohawn P. Anifrolumab, an anti-interferon-alpha receptor monoclonal antibody, in moderate-to-severe systemic lupus erythematosus. Arthritis Rheumatol (2017) 69(2):376–86. doi: 10.1002/art.39962 PMC5299497 28130918

[24] Morand EF Furie R Tanaka Y Bruce IN Askanase AD Richez C. Trial of anifrolumab in active systemic lupus erythematosus. N Engl J Med (2020) 382(3):211–21. doi: 10.1056/NEJMoa1912196 31851795

[25] Abrahamowicz M Fortin PR du Berger R Nayak V Neville C Liang MH. The relationship between disease activity and expert physician's decision to start major treatment in active systemic lupus erythematosus: A decision aid for development of entry criteria for clinical trials. J Rheumatol (1998) 25(2):277–84. 9489819

[26] Barr SG Zonana-Nacach A Magder LS Petri M. Patterns of disease activity in systemic lupus erythematosus. Arthritis Rheum (1999) 42(12):2682–8. 10.1002/1529-0131(199912)42:12<2682::AID-ANR26>3.0.CO;2-6 10616018

[27] Merrill JT Neuwelt CM Wallace DJ Shanahan JC Latinis KM Oates JC. Efficacy and safety of rituximab in moderately-to-severely active systemic lupus erythematosus: The randomized, double-blind, phase II/III systemic lupus erythematosus evaluation of rituximab trial. Arthritis Rheum (2010) 62(1):222–33. doi: 10.1002/art.27233 PMC4548300 20039413

[28] Rovin BH Furie R Latinis K Looney RJ Fervenza FC Sanchez-Guerrero J. Efficacy and safety of rituximab in patients with active proliferative lupus nephritis: The lupus nephritis assessment with rituximab study. Arthritis Rheum (2012) 64(4):1215–26. doi: 10.1002/art.34359 22231479

[29] McCarthy EM Sutton E Nesbit S White J Parker B Jayne D. Short-term efficacy and safety of rituximab therapy in refractory systemic lupus erythematosus: Results from the British Isles Lupus Assessment Group Biologics Register. Rheumatology (2018) 57(3):470–9. doi: 10.1093/rheumatology/kex395 PMC5850287 29216396

[30] Witt M Grunke M Proft F Baeuerle M Aringer M Burmester G. Clinical outcomes and safety of rituximab treatment for patients with systemic lupus erythematosus (SLE) - results from a nationwide cohort in Germany (Graid). Lupus (2013) 22(11):1142–9. doi: 10.1177/0961203313503912 24057058

[31] Terrier B Amoura Z Ravaud P Hachulla E Jouenne R Combe B. Safety and efficacy of rituximab in systemic lupus erythematosus: Results from 136 patients from the French autoimmunity and rituximab registry. Arthritis Rheum (2010) 62(8):2458–66. doi: 10.1002/art.27541 20506527

[32] Iaccarino L Bartoloni E Carli L Ceccarelli F Conti F De Vita S. Efficacy and safety of off-label use of rituximab in refractory lupus: Data from the Italian multicentre registry. Clin Exp Rheumatol (2015) 33(4):449–56. 26053285

[33] Iwata S Saito K Hirata S Ohkubo N Nakayamada S Nakano K. Efficacy and safety of anti-CD20 antibody rituximab for patients with refractory systemic lupus erythematosus. Lupus (2018) 27(5):802–11. doi: 10.1177/0961203317749047 29308726

[34] Fernandez-Nebro A de la Fuente JL Carreno L Izquierdo MG Tomero E Rua-Figueroa I. Multicenter longitudinal study of B-lymphocyte depletion in refractory systemic lupus erythematosus: The lesimab study. Lupus (2012) 21(10):1063–76. doi: 10.1177/0961203312446627 22786985

[35] Cassia MA Alberici F Jones RB Smith RM Casazza G Urban ML. Rituximab as maintenance treatment for systemic lupus erythematosus: A multicenter observational study of 147 patients. Arthritis Rheumatol (2019) 71(10):1670–80. doi: 10.1002/art.40932 31102498

[36] Hickman RA Hira-Kazal R Yee CS Toescu V Gordon C. The efficacy and safety of rituximab in a chart review study of 15 patients with systemic lupus erythematosus. Clin Rheumatol (2015) 34(2):263–71. doi: 10.1007/s10067-014-2839-0 25564308

[37] Santos JE Fiel D Santos R Vicente R Aguiar R Santos I. Rituximab use in adult glomerulopathies and its rationale. J Bras Nefrol (2020) 42(1):77–93. doi: 10.1590/2175-8239-jbn-2018-0254 31904761 PMC7213927

[38] Duxbury B Combescure C Chizzolini C. Rituximab in systemic lupus erythematosus: An updated systematic review and meta-analysis. Lupus (2013) 22(14):1489–503. doi: 10.1177/0961203313509295 24135078

[39] Alshaiki F Obaid E Almuallim A Taha R El-Haddad H Almoallim H. Outcomes of rituximab therapy in refractory lupus: A meta-analysis. Eur J Rheumatol (2018) 5(2):118–26. doi: 10.5152/eurjrheum.2018.17096 PMC6072690 30185361

[40] Zhong Z Li H Zhong H Zhou T. Clinical efficacy and safety of rituximab in lupus nephritis. Drug Des Devel Ther (2019) 13:845–56. doi: 10.2147/DDDT.S195113 PMC6417005 30880917

[41] Kotagiri P Martin A Hughes P Becker G Nicholls K. Single-dose rituximab in refractory lupus nephritis. Intern Med J (2016) 46(8):899–901. doi: 10.1111/imj.13136 27242250

[42] Moroni G Raffiotta F Trezzi B Giglio E Mezzina N Del Papa N. Rituximab vs mycophenolate and vs cyclophosphamide pulses for induction therapy of active lupus nephritis: A clinical observational study. Rheumatology (2014) 53(9):1570–7. doi: 10.1093/rheumatology/ket462 24505125

[43] Chavarot N Verhelst D Pardon A Caudwell V Mercadal L Sacchi A. Rituximab alone as induction therapy for membranous lupus nephritis: A multicenter retrospective study. Medicine (Baltimore) (2017) 96(27):e7429. doi: 10.1097/MD.0000000000007429 28682905 PMC5502178

[44] Diaz-Lagares C Croca S Sangle S Vital EM Catapano F Martinez-Berriotxoa A. Efficacy of rituximab in 164 patients with biopsy-proven lupus nephritis: Pooled data from European cohorts. Autoimmun Rev (2012) 11(5):357–64. doi: 10.1016/j.autrev.2011.10.009 22032879

[45] Zhou J Tao MJ Jin LR Sheng J Li Z Peng H. Effectiveness and safety of common therapeutic drugs for refractory lupus nephritis: A network meta-analysis. Exp Ther Med (2020) 19(1):665–71. doi: 10.3892/etm.2019.8257 PMC6923745 31897105

[46] Jiang B Li T Guo L Shen H Ye S Chen S. Efficacy and safety of rituximab in systemic lupus erythematosus and Sjögren syndrome patients with refractory thrombocytopenia: A retrospective study of 21 cases. J Clin Rheumatol (2015) 21(5):244–50. doi: 10.1097/RHU.0000000000000273 PMC4539196 26203828

[47] Serris A Amoura Z Canoui-Poitrine F Terrier B Hachulla E Costedoat-Chalumeau N. Efficacy and safety of rituximab for systemic lupus erythematosus-associated immune cytopenias: A multicenter retrospective cohort study of 71 adults. Am J Hematol (2018) 93(3):424–9. doi: 10.1002/ajh.24999 29247540

[48] Jovancevic B Lindholm C Pullerits R. Anti B-cell therapy against refractory thrombocytopenia in SLE and MCTD patients: Long-term follow-up and review of the literature. Lupus (2013) 22(7):664–74. doi: 10.1177/0961203313485489 23612795

[49] Pavo-Blanco M Novella-Navarro M Caliz-Caliz R Ferrer-Gonzalez MA. Rituximab in refractory autoimmune hemolytic anemia in systemic lupus erythematosus. Reumatol Clin (Engl Ed) (2018) 14(4):248–9. doi: 10.1016/j.reuma.2017.07.023 28870533

[50] Chao SH Chang YL Yen JC Liao HT Wu TH Yu CL. Efficacy and safety of rituximab in autoimmune and microangiopathic hemolytic anemia: A systematic review and meta-analysis. Exp Hematol Oncol (2020) 9:6. doi: 10.1186/s40164-020-00163-5 32322437 PMC7161265

[51] Abe K Ishikawa Y Ishikawa J Fujiwara M Kita Y. Successful treatment of a patient with refractory immune thrombocytopenic purpura in systemic lupus erythematosus with rituximab. Immunol Med (2019) 42(4):185–8. doi: 10.1080/25785826.2019.1696644 31794352

[52] Magro-Checa C Zirkzee EJ Huizinga TW Steup-Beekman GM. Management of neuropsychiatric systemic lupus erythematosus: Current approaches and future perspectives. Drugs (2016) 76(4):459–83. doi: 10.1007/s40265-015-0534-3 PMC4791452 26809245

[53] Tokunaga M Saito K Kawabata D Imura Y Fujii T Nakayamada S. Efficacy of rituximab (anti-CD20) for refractory systemic lupus erythematosus involving the central nervous system. Ann Rheum Dis (2007) 66(4):470–5. doi: 10.1136/ard.2006.057885 PMC1856059 17107983

[54] Narvaez J Rios-Rodriguez V de la Fuente D Estrada P Lopez-Vives L Gomez-Vaquero C. Rituximab therapy in refractory neuropsychiatric lupus: Current clinical evidence. Semin Arthritis Rheum (2011) 41(3):364–72. doi: 10.1016/j.semarthrit.2011.06.004 21875742

[55] Man BL Mok CC Fu YP. Neuro-ophthalmologic manifestations of systemic lupus erythematosus: A systematic review. Int J Rheum Dis (2014) 17(5):494–501. doi: 10.1111/1756-185X.12337 24673755

[56] Ye Y Qian J Gu Y Chen X Ye S. Rituximab in the treatment of severe lupus myelopathy. Clin Rheumatol (2011) 30(7):981–6. doi: 10.1007/s10067-011-1714-5 21340494

[57] Chessa E Piga M Floris A Mathieu A Cauli A. Severe neuropsychiatric systemic lupus erythematosus successfully treated with rituximab: An alternative to standard of care. Open Access Rheumatol (2017) 9:167–70. doi: 10.2147/OARRR.S143768 PMC5602683 28979169

[58] Angeletti A Baraldi O Chiocchini AL Comai G Cravedi P La Manna G. Rituximab as first-line therapy in severe lupus erythematosus with neuropsychiatric and renal involvement: A case-report and review of the literature. J Clin Case Rep (2017) 7(10):1033. doi: 10.4172/2165-7920.10001033 29888753 PMC5991483

[59] Gualtierotti R Borghi MO Gerosa M Schioppo T Larghi P Geginat J. Successful sequential therapy with rituximab and belimumab in patients with active systemic lupus erythematosus: A case series. Clin Exp Rheumatol (2018) 36(4):643–7. 29533753

[60] Kraaij T Arends EJ van Dam LS Kamerling SWA van Daele PLA Bredewold OW. Long-term effects of combined B-cell immunomodulation with rituximab and belimumab in severe, refractory systemic lupus erythematosus: 2-year results. Nephrol Dial Transplant (2021) 36(8):1474–83. doi: 10.1093/ndt/gfaa117 PMC8311580 32591783

[61] Kraaij T Kamerling SWA de Rooij ENM van Daele PLA Bredewold OW Bakker JA. The net-effect of combining rituximab with belimumab in severe systemic lupus erythematosus. J Autoimmun (2018) 91:45–54. doi: 10.1016/j.jaut.2018.03.003 29636274

[62] Kraaij T Huizinga TW Rabelink TJ Teng YK. Belimumab after rituximab as maintenance therapy in lupus nephritis. Rheumatology (2014) 53(11):2122–4. doi: 10.1093/rheumatology/keu369 25205827

[63] Simonetta F Allali D Roux-Lombard P Chizzolini C. Successful treatment of refractory lupus nephritis by the sequential use of rituximab and belimumab. Joint Bone Spine (2017) 84(2):235–6. doi: 10.1016/j.jbspin.2016.01.008 27238199

[64] Atisha-Fregoso Y Malkiel S Harris KM Byron M Ding L Kanaparthi S. phase II randomized trial of rituximab plus cyclophosphamide followed by belimumab for the treatment of lupus nephritis. Arthritis Rheumatol (2021) 73(1):121–31. doi: 10.1002/art.41466 PMC7839443 32755035

[65] Ichikawa T Shimojima Y Kishida D Kaneko T Sekijima Y. Primary central nervous system lymphoma in neuropsychiatric systemic lupus erythematosus: Case-based review. Rheumatol Int (2021) 41(5):1009–17. doi: 10.1007/s00296-020-04569-6 32253501

[66] Jones A Muller P Dore CJ Ikeji F Caverly E Chowdhury K. Belimumab after B cell depletion therapy in patients with systemic lupus erythematosus (BEAT-LUPUS) protocol: A prospective multicentre, double-blind, randomised, placebo-controlled, 52-week phase II clinical trial. BMJ Open (2019) 9(12):e032569. doi: 10.1136/bmjopen-2019-032569 PMC6937022 31848169

[67] Teng YKO Bruce IN Diamond B Furie RA van Vollenhoven RF Gordon D. phase III, multicentre, randomised, double-blind, placebo-controlled, 104-week study of subcutaneous belimumab administered in combination with rituximab in adults with systemic lupus erythematosus (SLE): BLISS-BELIEVE study protocol. BMJ Open (2019) 9(3):e025687. doi: 10.1136/bmjopen-2018-025687 PMC6475247 30898822

[68] Aranow C Allaart C Amoura Z. Efficacy and safety of subcutaneous belimumab (BEL) and rituximab (RTX) sequential therapy in patients with systemic lupus erythematosus: The phase 3, randomized, placebo-controlled BLISS-BELIEVE study. Arthritis Rheumatol (2021) 73(Suppl 9):L13. 10.1136/ard-2024-225686 PMC11503042 39159997

[69] Alunno A Padjen I Fanouriakis A Boumpas DT. Pathogenic and therapeutic relevance of Jak/Stat signaling in systemic lupus erythematosus: Integration of distinct inflammatory pathways and the prospect of their inhibition with an oral agent. Cells (2019) 8(8):898. doi: 10.3390/cells8080898 31443172 PMC6721755

[70] Wallace DJ Furie RA Tanaka Y Kalunian KC Mosca M Petri MA. Baricitinib for systemic lupus erythematosus: A double-blind, randomised, placebo-controlled, phase 2 trial. Lancet (2018) 392(10143):222–31. doi: 10.1016/S0140-6736(18)31363-1 30043749

[71] Yuan K Huang G Sang X Xu A. Baricitinib for systemic lupus erythematosus. Lancet (2019) 393(10170):402. doi: 10.1016/S0140-6736(18)32763-6 30712894

[72] Wallace DJ Furie RA Tanaka Y de Bono S Hoffman RW. Baricitinib for systemic lupus erythematosus - authors' reply. Lancet (2019) 393(10170):402–3. doi: 10.1016/S0140-6736(18)32749-1 30712893

[73] Alexander T Sarfert R Klotsche J Kuhl AA Rubbert-Roth A Lorenz HM. The proteasome inhibitior bortezomib depletes plasma cells and ameliorates clinical manifestations of refractory systemic lupus erythematosus. Ann Rheum Dis (2015) 74(7):1474–8. doi: 10.1136/annrheumdis-2014-206016 PMC4484251 25710470

[74] Zhang H Liu Z Huang L Hou J Zhou M Huang X. The short-term efficacy of bortezomib combined with glucocorticoids for the treatment of refractory lupus nephritis. Lupus (2017) 26(9):952–8. doi: 10.1177/0961203316686703 28059023

[75] Segarra A Arredondo KV Jaramillo J Jatem E Salcedo MT Agraz I. Efficacy and safety of bortezomib in refractory lupus nephritis: A single-center experience. Lupus (2020) 29(2):118–25. doi: 10.1177/0961203319896018 31865857

[76] Wang Y Zhou W Zhang Z. Successful treatment of warm-type haemolytic anaemia with bortezomib in a rituximab-failed systemic lupus erythematosus patient. Rheumatology (2015) 54(1):194–5. doi: 10.1093/rheumatology/keu393 25288787

[77] Quartuccio L Rupolo M Michieli M De Vita S. Efficacy and tolerability of repeated cycles of a once-weekly regimen of bortezomib in lupus. Rheumatology (2014) 53(2):381–2. doi: 10.1093/rheumatology/ket284 23962626

[78] Ishii T Tanaka Y Kawakami A Saito K Ichinose K Fujii H. Multicenter double-blind randomized controlled trial to evaluate the effectiveness and safety of bortezomib as a treatment for refractory systemic lupus erythematosus. Mod Rheumatol (2018) 28(6):986–92. doi: 10.1080/14397595.2018.1432331 29363990

[79] Sjowall C Hjorth M Eriksson P. Successful treatment of refractory systemic lupus erythematosus using proteasome inhibitor bortezomib followed by belimumab: Description of two cases. Lupus (2017) 26(12):1333–8. doi: 10.1177/0961203317691371 28162031

[80] Sciascia S Radin M Yazdany J Tektonidou M Cecchi I Roccatello D. Expanding the therapeutic options for renal involvement in lupus: Eculizumab, available evidence. Rheumatol Int (2017) 37(8):1249–55. doi: 10.1007/s00296-017-3686-5 28258475

[81] Furie R Matis L Rollins S Mojcik C. A single dose, placebo controlled, double blind, phase I study of the humanized anti-C5 antibody h5G1.1 in patients with systemic lupus erythematosus. Arthritis Rheum (2004) 50:S35–S747.

[82] Pickering MC Ismajli M Condon MB McKenna N Hall AE Lightstone L. Eculizumab as rescue therapy in severe resistant lupus nephritis. Rheumatology (2015) 54(12):2286–8. doi: 10.1093/rheumatology/kev307 PMC4643725 26316577

[83] Ono M Ohashi N Namikawa A Katahashi N Ishigaki S Tsuji N. A rare case of lupus nephritis presenting as thrombotic microangiopathy with diffuse pseudotubulization possibly caused by atypical hemolytic uremic syndrome. Intern Med (2018) 57(11):1617–23. doi: 10.2169/internalmedicine.0228-17 PMC6028688 29434134

[84] Wright RD Bannerman F Beresford MW Oni L. A systematic review of the role of eculizumab in systemic lupus erythematosus-associated thrombotic microangiopathy. BMC Nephrol (2020) 21(1):245. doi: 10.1186/s12882-020-01888-5 32605540 PMC7329551

[85] Talaat RM Mohamed SF Bassyouni IH Raouf AA. Th1/Th2/Th17/Treg cytokine imbalance in systemic lupus erythematosus (SLE) patients: Correlation with disease activity. Cytokine (2015) 72(2):146–53. doi: 10.1016/j.cyto.2014.12.027 25647269

[86] Satoh Y Nakano K Yoshinari H Nakayamada S Iwata S Kubo S. A case of refractory lupus nephritis complicated by psoriasis vulgaris that was controlled with secukinumab. Lupus (2018) 27(7):1202–6. doi: 10.1177/0961203318762598 29523055

[87] Illei GG Shirota Y Yarboro CH Daruwalla J Tackey E Takada K. Tocilizumab in systemic lupus erythematosus: Data on safety, preliminary efficacy, and impact on circulating plasma cells from an open-label phase I dosage-escalation study. Arthritis Rheum (2010) 62(2):542–52. doi: 10.1002/art.27221 PMC3057537 20112381

[88] Juptner M Zeuner R Schreiber S Laudes M Schroder JO. Successful application of belimumab in two patients with systemic lupus erythematosus experiencing a flare during tocilizumab treatment. Lupus (2014) 23(4):428–30. doi: 10.1177/0961203314520844 24482144

[89] Maeshima K Ishii K Torigoe M Imada C Iwakura M Hamasaki H. Successful tocilizumab and tacrolimus treatment in a patient with rheumatoid arthritis complicated by systemic lupus erythematosus. Lupus (2012) 21(9):1003–6. doi: 10.1177/0961203312441046 22433919

[90] Ocampo V Haaland D Legault K Mittoo S Aitken E. Successful treatment of recurrent pleural and pericardial effusions with tocilizumab in a patient with systemic lupus erythematous. BMJ Case Rep (2016) 2016:bcr2016215423. doi: 10.1136/bcr-2016-215423 PMC4986022 27503940

[91] Kamata Y Minota S. Successful treatment of massive intractable pericardial effusion in a patient with systemic lupus erythematosus with tocilizumab. BMJ Case Rep (2012) 2012:bcr2012007834. doi: 10.1136/bcr-2012-007834 PMC4544961 23264273

[92] Garcia-Hernandez FJ Gonzalez-Leon R Castillo-Palma MJ Ocana-Medina C Sanchez-Roman J. Tocilizumab for treating refractory haemolytic anaemia in a patient with systemic lupus erythematosus. Rheumatology (2012) 51(10):1918–9. doi: 10.1093/rheumatology/kes072 22513150

[93] Group AT. Treatment of lupus nephritis with abatacept: The abatacept and cyclophosphamide combination efficacy and safety study. Arthritis Rheumatol (2014) 66(11):3096–104. doi: 10.1002/art.38790 PMC4528976 25403681

[94] Furie R Nicholls K Cheng TT Houssiau F Burgos-Vargas R Chen SL. Efficacy and safety of abatacept in lupus nephritis: A twelve-month, randomized, double-blind study. Arthritis Rheumatol (2014) 66(2):379–89. doi: 10.1002/art.38260 24504810

[95] Merrill JT Burgos-Vargas R Westhovens R Chalmers A D'Cruz D Wallace DJ. The efficacy and safety of abatacept in patients with non-life-threatening manifestations of systemic lupus erythematosus: Results of a twelve-month, multicenter, exploratory, phase IIb, randomized, double-blind, placebo-controlled trial. Arthritis Rheum (2010) 62(10):3077–87. doi: 10.1002/art.27601 20533545

[96] Chamberlain C Colman PJ Ranger AM Burkly LC Johnston GI Otoul C. Repeated administration of dapirolizumab pegol in a randomised phase I study is well tolerated and accompanied by improvements in several composite measures of systemic lupus erythematosus disease activity and changes in whole blood transcriptomic profiles. Ann Rheum Dis (2017) 76(11):1837–44. doi: 10.1136/annrheumdis-2017-211388 28780512

[97] Furie RA Bruce IN Dörner T Leon MG Leszczyński P Urowitz M. Phase 2, randomized, placebo-controlled trial of dapirolizumab pegol in patients with moderate-to-severe active systemic lupus erythematosus. Rheumatology (2021) 60(11):5397–407. doi: 10.1093/rheumatology/keab381 PMC9194804 33956056

[98] Ostendorf L Burns M Durek P Heinz GA Heinrich F Garantziotis P. Targeting CD38 with daratumumab in refractory systemic lupus erythematosus. N Engl J Med (2020) 383(12):1149–55. doi: 10.1056/NEJMoa2023325 32937047

[99] Yalcin Mutlu M Wacker J Tascilar K Taubmann J Manger B Krönke G. Effective and safe treatment of anti-CD38 therapy in systemic lupus erythematosus–associated refractory cerebral vasculitis induces immune tolerance. Rheumatology (2023) 62(2):e21–e3. doi: 10.1093/rheumatology/keac393 35801920

[100] Mulhearn B Bruce IN. Indications for IVIG in rheumatic diseases. Rheumatol (Oxford England) (2015) 54(3):383–91. doi: 10.1093/rheumatology/keu429 PMC4334686 25406359

[101] Sakthiswary R D'Cruz D. Intravenous immunoglobulin in the therapeutic armamentarium of systemic lupus erythematosus: A systematic review and meta-analysis. Medicine (2014) 93(16):e86. doi: 10.1097/md.0000000000000086 25310743 PMC4616295

[102] Duarte AC Sousa S Nunes T Cordeiro A Gonçalves P. Intravenous human immunoglobulin for the treatment of severe longitudinal extensive transverse myelitis associated with systemic lupus erythematous. Acta Reumatol Port (2018) 43(2):154–5. 30091960

[103] Humrich JY Riemekasten G. Low-dose interleukin-2 therapy for the treatment of systemic lupus erythematosus. Curr Opin Rheumatol (2019) 31(2):208–12. doi: 10.1097/BOR.0000000000000575 30562181

[104] Aringer M Smolen JS. The role of tumor necrosis factor-alpha in systemic lupus erythematosus. Arthritis Res Ther (2008) 10(1):202. doi: 10.1186/ar2341 18226185 PMC2374473

[105] Aringer M Houssiau F Gordon C Graninger WB Voll RE Rath E. Adverse events and efficacy of TNF-alpha blockade with infliximab in patients with systemic lupus erythematosus: Long-term follow-up of 13 patients. Rheumatology (2009) 48(11):1451–4. doi: 10.1093/rheumatology/kep270 19748965

[106] Furie RA Aroca G Cascino MD Garg JP Rovin BH Alvarez A. B-cell depletion with obinutuzumab for the treatment of proliferative lupus nephritis: A randomised, double-blind, placebo-controlled trial. Ann Rheum Dis (2021) 81(1):100–7. doi: 10.1136/annrheumdis-2021-220920 PMC8762029 34615636

[107] Md Yusof MY Shaw D El-Sherbiny YM Dunn E Rawstron AC Emery P. Predicting and managing primary and secondary non-response to rituximab using B-cell biomarkers in systemic lupus erythematosus. Ann Rheum Dis (2017) 76(11):1829–36. doi: 10.1136/annrheumdis-2017-211191 PMC5705851 28684557

[108] Masoud S McAdoo SP Bedi R Cairns TD Lightstone L. Ofatumumab for B cell depletion in patients with systemic lupus erythematosus who are allergic to rituximab. Rheumatology (2018) 57(7):1156–61. doi: 10.1093/rheumatology/key042 29562252

[109] Mackensen A Müller F Mougiakakos D Böltz S Wilhelm A Aigner M. Anti-CD19 CAR T cell therapy for refractory systemic lupus erythematosus. Nat Med (2022) 28(10):2124–32. doi: 10.1038/s41591-022-02017-5 36109639

[110] Schreiber K Sciascia S de Groot PG Devreese K Jacobsen S Ruiz-Irastorza G. Antiphospholipid syndrome. Nat Rev Dis Primers (2018) 4:17103. doi: 10.1038/nrdp.2017.103 29321641

[111] Cervera R Serrano R Pons-Estel GJ Ceberio-Hualde L Shoenfeld Y de Ramón E. Morbidity and mortality in the antiphospholipid syndrome during a 10-year period: A multicentre prospective study of 1000 patients. Ann Rheum Dis (2015) 74(6):1011–8. doi: 10.1136/annrheumdis-2013-204838 24464962

[112] Serrano R Pons-Estel GJ Espinosa G Quintana RM Reverter JC Tassies D. Long-term follow-up of antiphospholipid syndrome: Real-life experience from a single center. Lupus (2020) 29(9):1050–9. doi: 10.1177/0961203320933009 32536318

[113] Kazzaz NM McCune WJ Knight JS. Treatment of catastrophic antiphospholipid syndrome. Curr Opin Rheumatol (2016) 28(3):218–27. doi: 10.1097/BOR.0000000000000269 PMC4958413 26927441

[114] Tektonidou MG Andreoli L Limper M Amoura Z Cervera R Costedoat-Chalumeau N. EULAR recommendations for the management of antiphospholipid syndrome in adults. Ann Rheum Dis (2019) 78(10):1296–304. doi: 10.1136/annrheumdis-2019-215213 PMC11034817 31092409

[115] Cervera R Rodríguez-Pintó I Colafrancesco S Conti F Valesini G Rosário C. 14th international congress on antiphospholipid antibodies task force report on catastrophic antiphospholipid syndrome. Autoimmun Rev (2014) 13(7):699–707. doi: 10.1016/j.autrev.2014.03.002 24657970

[116] Ruiz-Irastorza G Cuadrado MJ Ruiz-Arruza I Brey R Crowther M Derksen R. Evidence-based recommendations for the prevention and long-term management of thrombosis in antiphospholipid antibody-positive patients: Report of a task force at the 13th international congress on antiphospholipid antibodies. Lupus (2011) 20(2):206–18. doi: 10.1177/0961203310395803 21303837

[117] Legault K Schunemann H Hillis C Yeung C Akl EA Carrier M. Mcmaster RARE-bestpractices clinical practice guideline on diagnosis and management of the catastrophic antiphospholipid syndrome. J Thromb Haemostasis (2018) 16(8):1656–64. doi: 10.1111/jth.14192 29978552

[118] Tenti S Cheleschi S Guidelli GM Galeazzi M Fioravanti A. Intravenous immunoglobulins and antiphospholipid syndrome: How, when and why? A review of the literature. Autoimmun Rev (2016) 15(3):226–35. doi: 10.1016/j.autrev.2015.11.009 26656906

[119] Kew GS Cho J Lateef A. Microangiopathic antiphospholipid antibody-associated syndrome in a pregnant lady. Lupus (2017) 26(4):435–7. doi: 10.1177/0961203316659548 27694537

[120] Ruffatti A Favaro M Hoxha A Zambon A Marson P Del Ross T. Apheresis and intravenous immunoglobulins used in addition to conventional therapy to treat high-risk pregnant antiphospholipid antibody syndrome patients. A Prospective Study. J Reprod Immunol (2016) 115:14–9. doi: 10.1016/j.jri.2016.03.004 27088752

[121] Mar N Kosowicz R Hook K. Recurrent thrombosis prevention with intravenous immunoglobulin and hydroxychloroquine during pregnancy in a patient with history of catastrophic antiphospholipid syndrome and pregnancy loss. J Thromb Thrombolysis (2014) 38(2):196–200. doi: 10.1007/s11239-014-1061-x 24549974

[122] Watanabe N Yamaguchi K Motomura K Hisano M Sago H Murashima A. Combination therapy with anticoagulants, corticosteroids and intravenous immunoglobulin for women with severe obstetric antiphospholipid syndrome. Clin Exp Rheumatol (2014) 32(2):299–300. 24447427

[123] Deng S Shen J Ni J Gong Y Zhu H. Cutaneous gangrene of the arms and legs after cardiopulmonary resuscitation: A rare presentation of catastrophic antiphospholipid syndrome. Am J Emergency Med (2017) 35(1):191 e3– e5. doi: 10.1016/j.ajem.2016.06.086 27396539

[124] Rosenbaum AN Anavekar NS Ernste FC Mankad SV Le RJ Manocha KK. A case of catastrophic antiphospholipid syndrome: First report with advanced cardiac imaging using MRI. Lupus (2015) 24(12):1338–41. doi: 10.1177/0961203315587960 26014099

[125] Shi VJ Leventhal JS Mensah KA Galan A Choate KA. Cyanosis of the foot. Cutis (2017) 100(4):206. 29136053

[126] Martis N Blanchouin E Lazdunski R Lechtman S Robert A Hyvernat H. A therapeutic challenge: Catastrophic anti-phospholipid syndrome with diffuse alveolar haemorrhage. Immunologic Res (2015) 62(2):222–4. doi: 10.1007/s12026-015-8649-x 25906846

[127] Berman H Rodriguez-Pinto I Cervera R Morel N Costedoat-Chalumeau N Erkan D. Rituximab use in the catastrophic antiphospholipid syndrome: Descriptive analysis of the CAPS registry patients receiving rituximab. Autoimmun Rev (2013) 12(11):1085–90. doi: 10.1016/j.autrev.2013.05.004 23777822

[128] Horikoshi M Inokuma S Matsubara E Honda Y Okada R Kobuna M. Atypical subacute recurrence of catastrophic antiphospholipid syndrome in a Japanese female patient. Intern Med (2015) 54(22):2923–7. doi: 10.2169/internalmedicine.54.5150 26568011

[129] Yachoui R Sehgal R Amlani B Goldberg JW. Antiphospholipid antibodies-associated diffuse alveolar hemorrhage. Semin Arthritis Rheum (2015) 44(6):652–7. doi: 10.1016/j.semarthrit.2014.10.013 25481816

[130] Wang CR Liu MF. Rituximab usage in systemic lupus erythematosus-associated antiphospholipid syndrome: A single-center experience. Semin Arthritis Rheum (2016) 46(1):102–8. doi: 10.1016/j.semarthrit.2016.02.002 26992634

[131] Sciascia S Radin M Bazzan M Roccatello D. Novel diagnostic and therapeutic frontiers in thrombotic anti-phospholipid syndrome. Intern Emerg Med (2017) 12(1):1–7. doi: 10.1007/s11739-016-1596-2 28044251

[132] Dioszegi A Tarr T Nagy-Vincze M Nanasy-Vass M Veisz R Bidiga L. Microthrombotic renal involvement in an SLE patient with concomitant catastrophic antiphospholipid syndrome: The beneficial effect of rituximab treatment. Lupus (2018) 27(9):1552–8. doi: 10.1177/0961203318768890 29635999

[133] Gkogkolou P Ehrchen J Goerge T. Severe antiphospholipid antibody syndrome - response to plasmapheresis and rituximab. J Dermatol Treat (2017) 28(6):564–6. doi: 10.1080/09546634.2017.1282599 28084106

[134] Erkan D Vega J Ramon G Kozora E Lockshin MD. A pilot open-label phase II trial of rituximab for non-criteria manifestations of antiphospholipid syndrome. Arthritis Rheum (2013) 65(2):464–71. doi: 10.1002/art.37759 23124321

[135] Mormile I Granata F Punziano A de Paulis A Rossi FW. Immunosuppressive treatment in antiphospholipid syndrome: Is it worth it? Biomedicines (2021) 9(2):132. doi: 10.3390/biomedicines9020132 33535377 PMC7911562

[136] Tinti MG Carnevale V Inglese M Molinaro F Bernal M Migliore A. Eculizumab in refractory catastrophic antiphospholipid syndrome: A case report and systematic review of the literature. Clin Exp Med (2019) 19(3):281–8. doi: 10.1007/s10238-019-00565-8 31214910

[137] Brito-Zeron P Baldini C Bootsma H Bowman SJ Jonsson R Mariette X. Sjogren syndrome. Nat Rev Dis Primers (2016) 2(1):16047. doi: 10.1038/nrdp.2016.47 27383445

[138] Both T Dalm VASH van Hagen PM van Daele PLA. Reviewing primary Sjögren's syndrome: Beyond the dryness - from pathophysiology to diagnosis and treatment. Int J Med Sci (2017) 14(3):191–200. doi: 10.7150/ijms.17718 28367079 PMC5370281

[139] Carsons SE Vivino FB Parke A Carteron N Sankar V Brasington R. Treatment guidelines for rheumatologic manifestations of Sjögren's syndrome: Use of biologic agents, management of fatigue, and inflammatory musculoskeletal pain. Arthritis Care Res (Hoboken) (2017) 69(4):517–27. doi: 10.1002/acr.22968 27390247

[140] Fasano S Isenberg DA. Present and novel biologic drugs in primary Sjogren's syndrome. Clin Exp Rheumatol (2019) 37 Suppl 118(3):167–74. 31025931

[141] Price EJ Rauz S Tappuni AR Sutcliffe N Hackett KL Barone F. The British Society for Rheumatology guideline for the management of adults with primary Sjögren's syndrome. Rheumatology (2017) 56(10):e24–48. doi: 10.1093/rheumatology/kex166 28957550

[142] Brito-Zeron P Retamozo S Kostov B Baldini C Bootsma H De Vita S. Efficacy and safety of topical and systemic medications: A systematic literature review informing the EULAR recommendations for the management of Sjogren's syndrome. RMD Open (2019) 5(2):e001064. doi: 10.1136/rmdopen-2019-001064 31749986 PMC6827762

[143] Ramos-Casals M Brito-Zeron P Bombardieri S Bootsma H De Vita S Dorner T. EULAR recommendations for the management of Sjogren's syndrome with topical and systemic therapies. Ann Rheum Dis (2020) 79(1):3–18. doi: 10.1136/annrheumdis-2019-216114 31672775

[144] Devauchelle-Pensec V Mariette X Jousse-Joulin S Berthelot JM Perdriger A Puechal X. Treatment of primary Sjögren's syndrome with rituximab: A randomized trial. Ann Intern Med (2014) 160(4):233–42. doi: 10.7326/M13-1085 24727841

[145] Bowman SJ Everett CC O'Dwyer JL Emery P Pitzalis C Ng WF. Randomized controlled trial of rituximab and cost-effectiveness analysis in treating fatigue and oral dryness in primary Sjögren's syndrome. Arthritis Rheumatol (2017) 69(7):1440–50. doi: 10.1002/art.40093 28296257

[146] Souza FB Porfirio GJ Andriolo BN Albuquerque JV Trevisani VF. Rituximab effectiveness and safety for treating primary Sjögren's syndrome (pSS): Systematic review and meta-analysis. PloS One (2016) 11(3):e0150749. doi: 10.1371/journal.pone.0150749 26998607 PMC4801187

[147] Letaief H Lukas C Barnetche T Gaujoux-Viala C Combe B Morel J. Efficacy and safety of biological DMARDs modulating B cells in primary Sjögren's syndrome: Systematic review and meta-analysis. Joint Bone Spine (2018) 85(1):15–22. doi: 10.1016/j.jbspin.2017.06.004 28673789

[148] Flores-Chavez A Kostov B Solans R Fraile G Maure B Feijoo-Masso C. Severe, life-threatening phenotype of primary Sjögren's syndrome: Clinical characterisation and outcomes in 1580 patients (GEAS-SS registry). Clin Exp Rheumatol (2018) 36 Suppl 112(3):121–9. 30156546

[149] Pollard RP Pijpe J Bootsma H Spijkervet FK Kluin PM Roodenburg JL. Treatment of mucosa-associated lymphoid tissue lymphoma in Sjögren's syndrome: A retrospective clinical study. J Rheumatol (2011) 38(10):2198–208. doi: 10.3899/jrheum.110077 21844152

[150] Meijer JM Meiners PM Vissink A Spijkervet FK Abdulahad W Kamminga N. Effectiveness of rituximab treatment in primary Sjögren's syndrome: A randomized, double-blind, placebo-controlled trial. Arthritis Rheum (2010) 62(4):960–8. doi: 10.1002/art.27314 20131246

[151] De Vita S Quartuccio L Isola M Mazzaro C Scaini P Lenzi M. A randomized controlled trial of rituximab for the treatment of severe cryoglobulinemic vasculitis. Arthritis Rheum (2012) 64(3):843–53. doi: 10.1002/art.34331 22147661

[152] Gottenberg JE Cinquetti G Larroche C Combe B Hachulla E Meyer O. Efficacy of rituximab in systemic manifestations of primary Sjögren's syndrome: Results in 78 patients of the autoimmune and rituximab registry. Ann Rheum Dis (2013) 72(6):1026–31. doi: 10.1136/annrheumdis-2012-202293 23264337

[153] Seror R Sordet C Guillevin L Hachulla E Masson C Ittah M. Tolerance and efficacy of rituximab and changes in serum B cell biomarkers in patients with systemic complications of primary Sjögren's syndrome. Ann Rheum Dis (2007) 66(3):351–7. doi: 10.1136/ard.2006.057919 PMC1856024 16950808

[154] Ramos-Casals M Garcia-Hernandez FJ de Ramon E Callejas JL Martinez-Berriotxoa A Pallares L. Off-label use of rituximab in 196 patients with severe, refractory systemic autoimmune diseases. Clin Exp Rheumatol (2010) 28(4):468–76. 20525449

[155] Figueiras M Sousa F Brandão M Oliveira D Faria R Campar A. P117 rituximab therapy for primary Sjögren's syndrome – a retrospective single-centre study. Lupus Sci Med (2020) 7(Suppl 1):A85–A. doi: 10.1136/lupus-2020-eurolupus.161

[156] De Vita S Quartuccio L Seror R Salvin S Ravaud P Fabris M. Efficacy and safety of belimumab given for 12 months in primary Sjögren's syndrome: The BELISS open-label phase II study. Rheumatology (2015) 54(12):2249–56. doi: 10.1093/rheumatology/kev257 26242856

[157] Mariette X Seror R Quartuccio L Baron G Salvin S Fabris M. Efficacy and safety of belimumab in primary Sjögren's syndrome: Results of the BELISS open-label phase II study. Ann Rheum Dis (2015) 74(3):526–31. doi: 10.1136/annrheumdis-2013-203991 24347569

[158] Machado AC Dos Santos LC Fidelix T Lekwitch I Soares SB Gasparini AF. Effectiveness and safety of abatacept for the treatment of patients with primary Sjögren's syndrome. Clin Rheumatol (2020) 39(1):243–8. doi: 10.1007/s10067-019-04724-w 31420813

[159] Baer AN Gottenberg JE St Clair EW Sumida T Takeuchi T Seror R. Efficacy and safety of abatacept in active primary Sjögren's syndrome: Results of a phase III, randomised, placebo-controlled trial. Ann Rheum Dis (2020) 80(3):339–48. doi: 10.1136/annrheumdis-2020-218599 PMC7892395 33168545

[160] van Nimwegen JF Mossel E van Zuiden GS Wijnsma RF Delli K Stel AJ. Abatacept treatment for patients with early active primary Sjögren's syndrome: A single-centre, randomised, double-blind, placebo-controlled, phase 3 trial (ASAP-III study). Lancet Rheumatol (2020) 2(3):e153–e63. doi: 10.1016/S2665-9913(19)30160-2 38263653

[161] Fanouriakis A Tziolos N Bertsias G Boumpas DT. Update on the diagnosis and management of systemic lupus erythematosus. Ann Rheumatic Dis (2021) 80(1):14–25. doi: 10.1136/annrheumdis-2020-218272 33051219

[162] Gazitt T Eviatar T Shear J Meidan R Furer V Feld J. Development of autoantibodies following BNT162b2 mRNA COVID-19 vaccination and their association with disease flares in adult patients with autoimmune inflammatory rheumatic diseases (AIIRD) and the general population: Results of 1-year prospective follow-up study. Vaccines (Basel) (2023) 11(2):476. doi: 10.3390/vaccines11020476 36851352 PMC9958930

[163] Murdaca G Orsi A Spanò F Puppo F Durando P Icardi G. Influenza and pneumococcal vaccinations of patients with systemic lupus erythematosus: Current views upon safety and immunogenicity. Autoimmun Rev (2014) 13(2):75–84. doi: 10.1016/j.autrev.2013.07.007 24044940

[164] Garg M Mufti N Palmore TN Hasni SA. Recommendations and barriers to vaccination in systemic lupus erythematosus. Autoimmun Rev (2018) 17(10):990–1001. doi: 10.1016/j.autrev.2018.04.006 30103044 PMC6150817

