# Supplementary material for: Biological therapy in systemic lupus erythematosus, antiphospholipid syndrome, and Sjögren’s syndrome: evidence- and practice-based guidance
Source: Front Immunol. 2023 Apr 17;14:1117699. doi: 10.3389/fimmu.2023.1117699 (PMC10150407; doi:10.3389/fimmu.2023.1117699)
Supplement: Supplementary file 1 [file DataSheet_1.docx]

Supplementary Material

# Supplementary Data

# PUBMED search criteria:

# (DISEASE AND ((infliximab) OR (etanercept) OR (adalimumab) OR (golimumab) OR (certolizumab) OR (daclizumab) OR (basiliximab) OR (eculizumab) OR (IL-2) OR (anakinra) OR (rilonacept) OR (canakinumab) OR (ustekinumab) OR (secukinumab) OR (briakinumab) OR (tocilizumab) OR (siltuximab) OR (sifalimumab) OR (anifrolumab) OR (rontalizumab) OR (mepolizumab) OR (rituximab) OR (ocrelizumab) OR (ofatumumab) OR (obinutuzumab) OR (epratuzumab) OR (inotuzumab) OR (belimumab) OR (atacicept) OR (blisibimod) OR (tabalumab) OR (abatacept) OR (bortezomib) OR (ruxolitinib) OR (tofacitinib) OR (oclacitinib) OR (baricitinib) OR (filgotinib) OR (gandotinib) OR (lestaurtinib) OR (momelotinib) OR (pacritinib) OR (upadacitinib) OR (peficitinib) OR (fedratinib) OR (irinotecan) OR (IVIG) OR (anti-CD40L) OR (Gevokizumab) OR (omalizumab) OR (apremilast) OR (Interferon alpha-2 [MeSH])),

# where DISEASE corresponded to each disease of interest – e.g., (Lupus Erythematosus, Systemic [MeSH]), (“Antiphospholipid syndrome” [MeSH]) or to ("Sjogren's Syndrome" [MeSH]). Other PubMed filters were added, namely publication dates (from 2014/01/01 to 2019/03/25), species (Humans), ages (adults 18+) and languages (English).

# Supplementary Figures and Tables

## Supplementary Figures


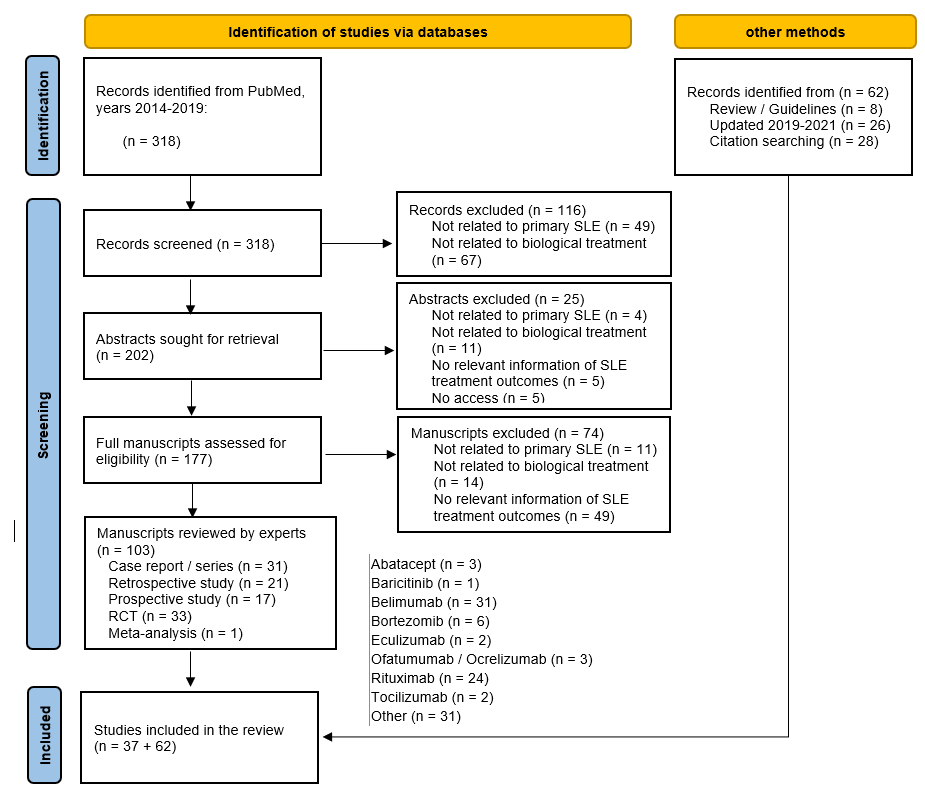


**Supplementary Figure 1.** Literature review for biologic therapy in systemic lupus erythematosus – PRISMA flow diagram


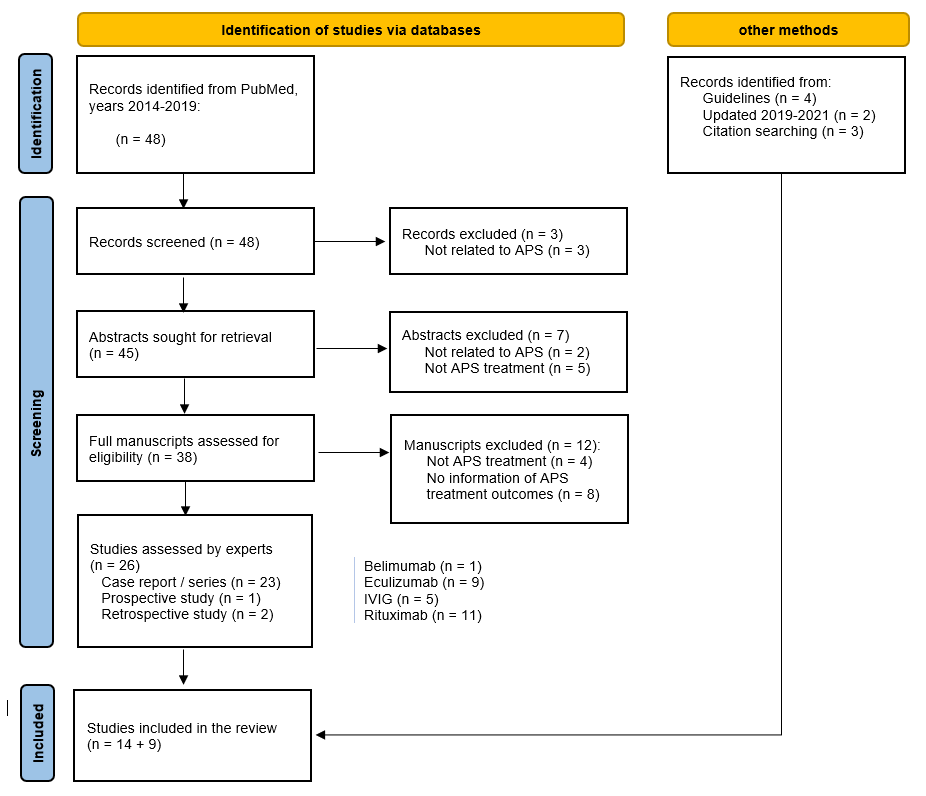


**Supplementary Figure 2.** Literature review for biologic therapy in antiphospholipid syndrome – PRISMA flow diagram


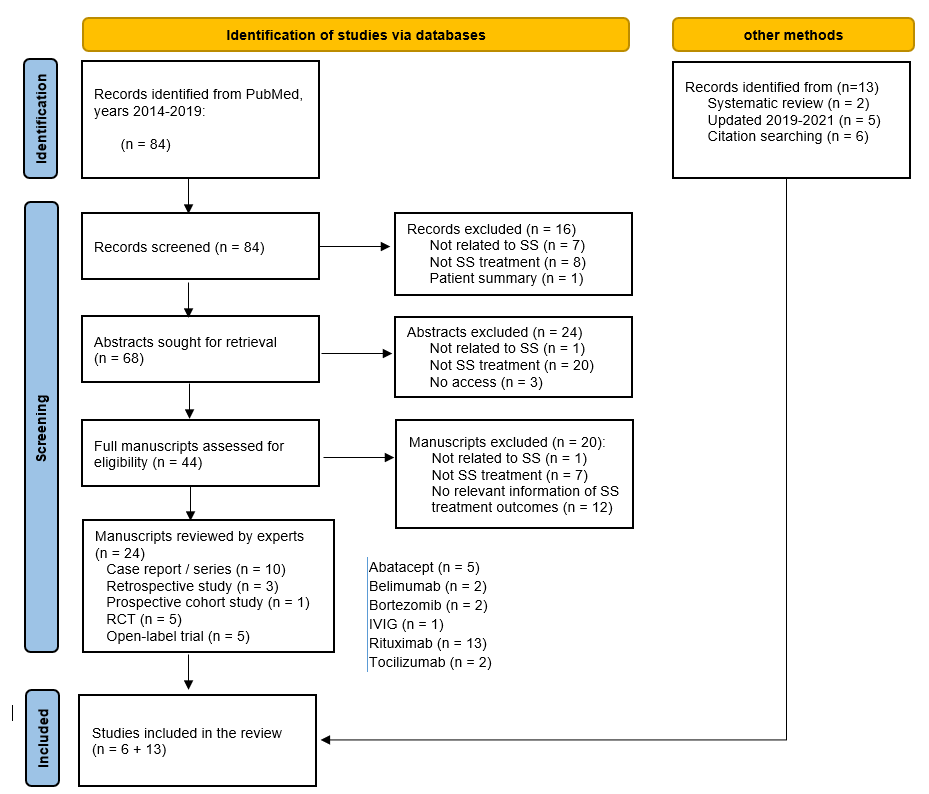


**Supplementary Figure 3.** Literature review for biologic therapy in Sjögren syndrome – PRISMA flow diagram

## Supplementary Tables

**Supplementary Table S1**. Levels of evidence and strength of recommendations

| Levels | Type of evidence | Strength | Evidence-based |
| --- | --- | --- | --- |
| 1a | Systematic review of RCTs | A | Consistent level 1 studies |
| 1b | Individual RCT |  |  |
| 2a | Systematic review of cohort studies | B | Consistent level 2 or 3 studies |
| 2b | Individual cohort study |  | Extrapolations from level 1 studies |
| 2c | Outcomes research / ecological studies |  |  |
| 3a | Systematic review of case-control studies |  |  |
| 3b | Individual case-control study |  |  |
| 4 | Case-series / retrospective studies / low-quality cohort or case control studies* | C | Consistent level 4 studies  Extrapolations from level 2 or 3 studies |
| 5 | Expert opinion | D | Consistent level 5 evidence  Troublingly inconsistent or inconclusive studies of any level |

* low quality defined according to the Oxford Centre for Evidence-based Medicine – Levels of Evidence (March 2009)

**Supplementary Table S2.** Consensus Results of Recommendations for off-label biologic therapy of Systemic Lupus Erythematosus.

| **Recommendation** (clinical circumstance and drug) | **LoE^a^** | **GoR^a^** | **Off-label guidance^b^** | **Consensus** | | | |
| --- | --- | --- | --- | --- | --- | --- | --- |
|  |  |  |  | **n/N** | **% ^c^** | **Decision** |  |
| **First-line therapy** | | | | | | | |
| 1. In pts with very active disease (i.e., SLEDAI>20 or BILAG 3A’s) |  |  |  |  |  |  |  |
| RTX is recommended | 2a | B | I | 15/16 | 94% | Approved |  |
| RTX-BEL may be used | 2b | B | II | 16/16 | 100% | Approved |  |
| 1. In pts with severe hemolytic anemia or severe thrombocytopenia (i.e., risk of death or organ damage), RTX is recommended | 4 | C | I | 15/16 | 94% | Approved |  |
| 1. In pts with HLH and ferritin > 10000 ng/mL, rapid progression, or organ damage, |  |  |  |  |  | Remove, due to the specificity of HLH and evolving treatment options |  |
| anakinra is recommended | 4 | C | I | 8/16 | 50% |  |  |
| 1. In pts with severe kidney disease (stage IV, presence of glomerular crescents and/or renal failure [GFR < 60 ml/min/1.73 m^2^]) |  |  |  |  |  |  |  |
| RTX is recommended | 2a | C | I | 15/16 | 94% | Approved |  |
| RTX-BEL may be used | 2b | C | II | 16/16 | 100% | Approved |  |
| 1. In pts with severe CNS disease |  |  |  |  |  |  |  |
| RTX is recommended | 4 | C | I | 15/16 | 94% | Approved |  |
| RTX-BEL may be used in recurrent cases | 4 | C | II | 16/16 | 100% | Approved |  |
| **Second-line therapy** | | | | | | | |
| 1. In pts with persistently active disease for at least one year, with flares |  |  |  |  |  |  |  |
| RTX is recommended in RTX-naïve cases | 2a | C | I | 16/16 | 100% | Approved |  |
| baricitinib may be used in pts with predominant arthritis flares | 1b | B | II | 16/16 | 100% | Approved |  |
| abatacept can be considered in pts with predominant arthritis flares | 1b | C | III | 8/16 | 50% | Remove |  |
| tocilizumab can be considered in pts with predominant arthritis flares | 4 | C | III | 14/16 | 88% | Approved |  |
| aTNF can be considered in pts with predominant arthritis flares | 2b | D | III | 1/16 | 6% | Remove |  |
| 1. In pts with severe kidney disease, |  |  |  |  |  |  |  |
| RTX is recommended in RTX-naïve cases | 2a | C | I | 16/16 | 100% | Approved |  |
| RTX-BEL may be used in multi-refractory cases | 4 | C | II | 16/16 | 100% | Approved |  |
| secukinumab can be considered in multi-refractory pts | 4 | D | III | 13/16 | 81% | Approved |  |
| eculizumab can be considered in multi-refractory pts | 4 | D | III | 1/16 | 6% | Remove |  |
| 1. In pts with very active disease, |  |  |  |  |  |  |  |
| RTX is recommended in RTX-naïve cases | 2a | B | I | 16/16 | 100% | Approved |  |
| RTX-BEL may be used in RTX-naïve cases | 2b | B | I | 16/16 | 100% | Approved |  |
| obinutuzumab may be used in multi‐refractory pts | 1b | B | II | 11/16 | 69% | Remove |  |
| ofatumumab can be considered in multi‐refractory pts | 4 | C | II | 4/16 | 25% | Remove |  |
| bortezomib can be considered in multi‐refractory pts | 4 | D | III | 15/16 | 94% | Approved |  |
| ocrelizumab can be considered if the previous response to RTX | 4 | C | IV | 2/16 | 13% | Remove |  |
| 1. In pts with hemolytic anemia or thrombocytopenia, |  |  |  |  |  |  |  |
| RTX is recommended in RTX-naïve cases | 4 | C | I | 16/16 | 100% | Approved |  |
| bortezomib can be considered in multi‐refractory cases | 4 | D | III | 16/16 | 100% | Approved |  |
| 1. In pts with hemophagocytic lymphohistiocytosis (HLH), |  |  |  |  |  | Remove, due to the specificity of HLH and evolving options |  |
| anakinra is recommended in ANAK-naïve cases | 4 | C | I | 8/16 | 50% |  |  |
| tocilizumab may be used in ANAK-experienced cases | 4 | C | II | 2/16 | 13% |  |  |
| 1. In pts with moderate or severe CNS disease, |  |  |  |  |  |  |  |
| RTX is recommended in RTX-naïve cases | 4 | C | I | 16/16 | 100% | Approved |  |
| RTX-BEL may be used in multi-refractory cases | 4 | D | II | 16/16 | 100% | Approved |  |
| *Abbreviations*: LoE, Level of Evidence. GoR, Grade of Recommendation. ANAK, anakinra. aTNF, anti-tumor necrosis factor. BILAG, British Isles Lupus Assessment Group index. CNS, Central Nervous System. GFR, Glomerular Filtration Rate. HLH, hemophagocytic lymphohistiocytosis. pts, patients. RTX, rituximab. RTX-BEL, rituximab, and belimumab (sequential therapy). SLEDAI, Systemic Lupus Erythematosus Disease Activity Index.  ^a^ LoE and GoR according to the Oxford CEMBE 2009 guidelines (see Table S1)  ^b^ Guidance of off-label use of biologic therapy, as defined by experts (see Table 1).  ^c^ Consensus (%): percentage of expert panel members that agreed with the recommendation. | | | | | | | |

**Supplementary Table S3.** Consensus results of recommendations for off-label biologic therapy of antiphospholipid syndrome.

| **Recommendation** (clinical circumstance and drug) | **LoE^a^** | **GoR^a^** | **Off-label guidance^b^** | **Consensus** | | | |
| --- | --- | --- | --- | --- | --- | --- | --- |
|  |  |  |  | **n/N** | **% ^c^** | **Decision** |  |
| **First-line therapy** | | | | | | | |
| 1. RTX is recommended as first-line therapy for APS patients with severe thrombocytopenia. | 2b | B | I | 15/16 | 94% | Approved |  |
| 1. The SoC in CAPS consists of combined therapy with anticoagulants, corticosteroids, plasmapheresis, or IVIG and systemic antibiotics if adequate. | 2b | D | I | 16/16 | 100% | Approved |  |
| 1. In patients with CAPS, RTX may be added to the combined therapy. | 2b | D | II | 16/16 | 100% | Approved |  |
| 1. In patients with CAPS and other SAIDs (e.g., SLE), RTX may be added to combined therapy. | 4 | D | II | 16/16 | 100% | Approved |  |
| **Second-line therapy** | | | | | | | |
| 1. As second-line therapy, the evidence available is insufficient to support any recommendations. | - | - | - | 16/16 | 100% | Approved |  |
| *Abbreviations*: LoE, Level of Evidence. GoR, Grade of Recommendation. APS, antiphospholipid syndrome. CAPS, catastrophic antiphospholipid syndrome. IVIG, intravenous immunoglobulin. RTX, rituximab. SAIDs, systemic autoimmune diseases. SLE, systemic lupus erythematosus. SoC, standard of care.  ^a^ LoE and GoR according to the Oxford CEMBE 2009 guidelines (see Table S1)  ^b^ Guidance of off-label use of biologic therapy, as defined by experts (see Table 1).  ^c^ Consensus (%): percentage of expert panel members that agreed with the recommendation. | | | | | | | |

**Supplementary Table S4.** Consensus results of recommendations for off-label biologic therapy in Sjögren’s syndrome.

| **Recommendation** (clinical circumstance and drug) | **LoE^a^** | **GoR^a^** | **Off-label guidance^b^** | **Consensus** | | | |
| --- | --- | --- | --- | --- | --- | --- | --- |
|  |  |  |  | **n/N** | **% ^c^** | **Decision** |  |
| **First-line therapy** | | | | | | | |
| 1. In pts with pSS and with sicca symptoms only, biological therapy is not recommended. | 1a | A | IV | 16/16 | 100% | Approved |  |
| 1. In pts with pSS and severe systemic manifestation, with risk of lymphoma (at least 3 risk factors for lymphoma) and recent onset (<12 months of evolution), RTX can be used as first-line therapy*.* | 4 | C | II | 16/16 | 100% | Approved |  |
| 1. RTX may be used as first-line therapy in pts with pSS (<12 months of evolution) and peripheral neuropathy, severe thrombocytopenia, severe CNS disease, severe parotid swelling, and/or cryoglobulinemic vasculitis. | 1b | B | II | 16/16 | 100% | Approved |  |
| 1. RTX may be used as first-line therapy in pts with pSS (>12 months of evolution) and peripheral neuropathy, severe thrombocytopenia, severe CNS disease, severe parotid swelling, and/or cryoglobulinemic vasculitis. | 2b | C | II | 16/16 | 100% | Approved |  |
| 1. Fatigue in pSS patients is not a recommendation for biological therapy. | 1a | A | IV | 16/16 | 100% | Approved |  |
| **Second-line therapy** | | | | | | | |
| 1. RTX is recommended in pts with refractory pSS and presenting systemic manifestations. | 1b | B | I | 16/16 | 100% | Approved |  |
| 1. In RTX-experienced pts, sequential therapy of BEL-RTX may be used. | 2b | C | II | 16/16 | 100% | Approved |  |
| 1. RTX can be considered for treatment of xerostomia in pts with refractory pSS and some residual salivary production and significant oral damage. | 1b | D | III | 2/16 | 13% | Remove from recommendations |  |
| *Abbreviations*: LoE, Level of Evidence. GoR, Grade of Recommendation. BEL, belimumab. CNS, central nervous system. pSS, primary Sjögren syndrome. pts, patients. RTX, rituximab.  ^a^ LoE and GoR according to the Oxford CEMBE 2009 guidelines (see Table S1)  ^b^ Guidance of off-label use of biologic therapy, as defined by experts (see Table 1).  ^c^ Consensus (%): percentage of expert panel members that agreed with the recommendation. | | | | | | | |
